# Aircraft-based inversions quantify the importance of wetlands and livestock for Upper Midwest methane emissions

**DOI:** 10.5194/acp-21-951-2021

**Published:** 2021-01-25

**Authors:** Xueying Yu, Dylan B. Millet, Kelley C. Wells, Daven K. Henze, Hansen Cao, Timothy J. Griffis, Eric A. Kort, Genevieve Plant, Malte J. Deventer, Randall K. Kolka, D. Tyler Roman, Kenneth J. Davis, Ankur R. Desai, Bianca C. Baier, Kathryn McKain, Alan C. Czarnetzki, A. Anthony Bloom

**Affiliations:** 1 Department of Soil, Water, and Climate, University of Minnesota, Saint Paul, Minnesota 55108, United States; 2 Department of Mechanical Engineering, University of Colorado Boulder, Boulder, Colorado 80309, United States; 3 Climate and Space Sciences and Engineering Department, University of Michigan, Ann Arbor, Michigan 48109, United States; 4 ANECO Institut für Umweltschutz GmbH & Co, 21079 Hamburg, Germany; 5 Northern Research Station, US Department of Agriculture Forest Service, Grand Rapids, Minnesota 55744, United States; 6 Department of Meteorology, The Pennsylvania State University, University Park, Pennsylvania 16802, United States; 7 Department of Atmospheric and Oceanic Sciences, University of Wisconsin-Madison, Madison, Wisconsin 53706, United States; 8 Cooperative Institute for Research in Environmental Sciences, University of Colorado, Boulder, Colorado 80309, United States; 9 Global Monitoring Laboratory, National Oceanic and Atmospheric Administration, Boulder, Colorado 80305, United States; 10 Department of Earth and Environmental Sciences, University of Northern Iowa, Cedar Falls, Iowa 50614, United States; 11 Jet Propulsion Laboratory, California Institute of Technology, Pasadena, California 91109, United States

## Abstract

We apply airborne measurements across three seasons (summer, winter and spring 2017–2018) in a multi-inversion framework to quantify methane emissions from the US Corn Belt and Upper Midwest, a key agricultural and wetland source region. Combing our seasonal results with prior fall values we find that wetlands are the largest regional methane source (32 %, 20 [16–23] Gg/d), while livestock (enteric/manure; 25 %, 15 [14–17] Gg/d) are the largest anthropogenic source. Natural gas/petroleum, waste/landfills, and coal mines collectively make up the remainder. Optimized fluxes improve model agreement with independent datasets within and beyond the study timeframe. Inversions reveal coherent and seasonally dependent spatial errors in the WetCHARTs ensemble mean wetland emissions, with an underestimate for the Prairie Pothole region but an overestimate for Great Lakes coastal wetlands. Wetland extent and emission temperature dependence have the largest influence on prediction accuracy; better representation of coupled soil temperature–hydrology effects is therefore needed. Our optimized regional livestock emissions agree well with the Gridded EPA estimates during spring (to within 7 %) but are ∼25 % higher during summer and winter. Spatial analysis further shows good top-down and bottom-up agreement for beef facilities (with mainly enteric emissions) but larger (∼30 %) seasonal discrepancies for dairies and hog farms (with >40 % manure emissions). Findings thus support bottom-up enteric emission estimates but suggest errors for manure; we propose that the latter reflects inadequate treatment of management factors including field application. Overall, our results confirm the importance of intensive animal agriculture for regional methane emissions, implying substantial mitigation opportunities through improved management.

## 1 Introduction

Atmospheric methane (CH_4_) has increased global radiative forcing by 0.97W/m^2^ since 1750 (IPCC, 2013), making it the most important anthropogenic greenhouse gas after carbon dioxide (CO_2_). Methane concentrations stabilized during the 1990s but resumed their increasing trend post-2007, with unclear causation (Kirschke et al., 2013; McNorton et al., 2018; Saunois et al., 2016; Thompson et al., 2018; Turner et al., 2017, 2019; Dlugokencky et al., 2011). Prior work suggests that US emission increases account for 20 %–60 % of the renewed global methane growth rate, with trends especially large in the central US (Alvarez et al., 2018; Franco et al., 2016; Helmig et al., 2016; Maasakkers et al., 2019; Sheng et al., 2018a; Turner et al., 2016). Quantifying emissions in this area is thus crucial for understanding the North American methane budget and its role in driving global trends. Here we employ new measurements from the GEM (Greenhouse Emissions in the Midwest) aircraft campaign in a multi-inversion framework to develop constraints on methane emissions from the Upper Midwest region.

Recent studies imply uncertainties in the magnitude and distribution of North American methane emissions (Kirschke et al., 2013; Miller and Michalak, 2017; Dlugokencky et al., 2011). For example, Turner et al. (2015) found, based on measurements from the Greenhouse Gases Observing SATellite (GOSAT), that the aggregated 2009–2011 US flux is 1.6× too low in the Emissions Database for Global Atmospheric Research (EDGAR v4.2, 2011; estimated US source of 26Tg/yr). However, subsequent work also using GOSAT retrievals (Maasakkers et al., 2019) concluded that the US flux is well-represented in the more recent Gridded Environmental Protection Agency inventory (GEPA Maasakkers et al., 2016) – with a US flux just 12 % higher than that of EDGAR v4.2 – and argued that the inferred EDGAR biases may instead reflect spatial errors in that inventory. Surface and aircraft-based inversion studies have further pointed to EPA bottom-up underestimates both nationally (Miller et al., 2013; Kort et al., 2008; Xiao et al., 2008; Karion et al., 2013; Wecht et al., 2014; Caulton et al., 2014) and regionally (Chen et al., 2018).

Wetlands are thought to be the single largest North American methane source (∼30 % of the total flux; Turner et al., 2015), but there are major uncertainties in the magnitude and spatiotemporal distribution of these emissions (Melton et al., 2013; Wania et al., 2013; Bruhwiler et al., 2014). For example, recent studies suggest an overestimate of wetland fluxes in Canada and the southeastern US (Miller et al., 2016; Sheng et al., 2018b) and that western Canadian and northern US wetland emissions have a broader spatial distribution than is predicted by models (Miller et al., 2014). Northern wetland emissions have strong seasonality, with a typical onset in late spring, peak in July–August, and decline in the fall with the onset of freezing. Bottom-up models have been shown to both underpredict and overpredict the width of this seasonal emission window, depending on location (Pickett-Heaps et al., 2011; Pugh et al., 2018; Knox et al., 2019; Peltola et al., 2019).

Livestock are the second-largest North American methane source, accounting for an estimated ∼25 % of the total continental flux (∼35 % of the anthropogenic flux) during 2009–2011 (Turner et al., 2015). However, enteric and manure emissions vary strongly with animal type, diet, management, and environmental factors (Niu et al., 2018; Charmley et al., 2016; Montes et al., 2013; Grant et al., 2015; Lassey, 2007; VanderZaag et al., 2014), and top-down studies have revealed large uncertainties in the resulting source estimates. For example, analyses of space-based, aircraft, and tall tower observations (Wecht et al., 2014; Miller et al., 2013) imply a 40 %–100 % underestimate of North American livestock emissions in the EDGAR v4.2 and 2013 EPA inventories. Tall tower measurements similarly point to a 1.8-fold GEPA livestock emission underestimate for the US Midwest (Chen et al., 2018). Space-based methane retrievals from GOSAT imply that US emissions rose by ∼20 % between 2010 and 2016, with a possible contribution from growing Midwest swine manure emissions (Sheng et al., 2018a). Previous studies have also revealed uncertainties in the spatial allocation of US livestock methane emissions: the spatial *R*^2^ between EDGAR v4.2 FT2010 and GEPA is only 0.5 for enteric fermentation and 0.1 for manure management, with the mismatch for the latter most significant in the Upper Midwest (Hristov et al., 2017). A facility-based analysis of concentrated animal feeding operations in this area based on GEM airborne data likewise pointed to spatial and temporal errors in bottom-up manure emissions (Yu et al., 2020).

The Upper Midwest is a crucial region for atmospheric methane: its extensive wetlands and > 700 million livestock (USDA-NASS, 2018) have been estimated to account for 30 % and 35 % of the total North American methane flux from wetlands and animal agriculture, respectively (Maasakkers et al., 2016; Bloom et al., 2017). The GEM study included extensive aircraft-based measurements of methane and related species across the Upper Midwest during three seasons (August 2017, January 2018, and May–June 2018, Fig. 1). The airborne sampling targeted wetland and agriculture emissions in particular, affording a unique opportunity to advance understanding of these sources. Here, we employ high-resolution chemical transport modeling (GEOS-Chem chemical transport model (CTM) at 0.25° × 0.3125°) in a multi-inversion framework (combining sector-based, Gaussian Mixture Model and adjoint 4D-Var analyses) to interpret the GEM datasets in terms of regional methane sources, with a focus on livestock and wetlands.

## 2 Data and methods

### 2.1 GEM flights and measurement payload

**Figure 1.**
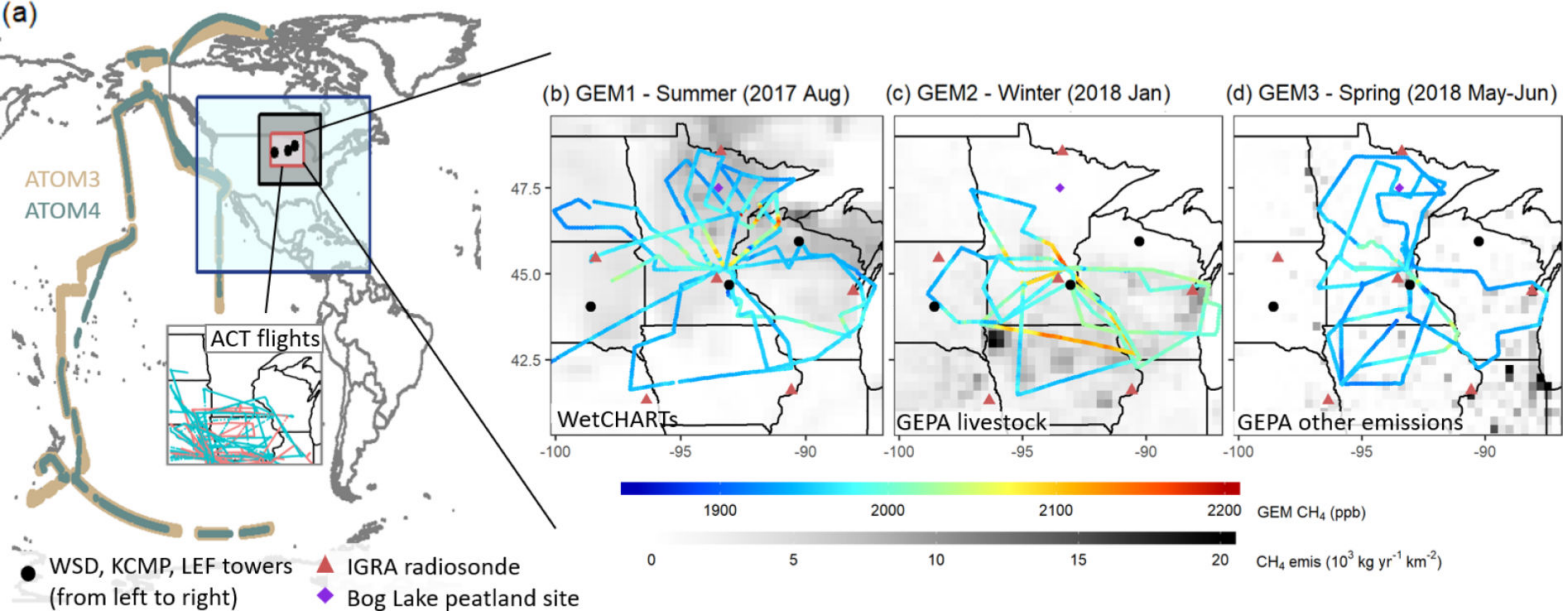
GEM flight tracks and additional datasets used in this study. Panel (**a**) shows Pacific flight tracks for the ATom3 and ATom4 campaigns used for evaluating modeled boundary and initial conditions. Also shown are the ACT-America flight tracks (C130 in cyan, B200 in red) used here for posterior model evaluation. The inner red box (40–50° N, 87–100° W) shows the GEM flight region that is expanded in (**b–d**), the black box (35–55° N, 80–105° W) shows the Upper Midwest analysis region employed for source inversions, and the blue box (9.75–60° N, 60–130° W) demarks the GEOS-Chem nested North American domain. The right panels show the GEM flight tracks colored by observed methane mixing ratios and superimposed on the prior annual bottom-up emissions described in-text. Also shown are locations for the radiosonde launches and tall towers employed here, along with the Bog Lake peatland eddy flux site.

The GEM aircraft campaign was designed to survey regional methane sources via downwind and upwind transects. Figure 1 shows sampling tracks, including 23 flights (156h) across three seasons (GEM1: 8 flights, 12–24 August 2017; GEM2: 7 flights, 17–28 January 2018; GEM3: 8 flights, 21 May–2 June 2018). Flights ranged from 4–8h in duration (mean: 6h) and took place in the daytime mixed layer (between 10:00 and 19:00 local standard time, 200–600ma.g.l.) onboard a Mooney aircraft with ∼280km/h boundary layer cruise speed (Scientific Aviation Inc.). Tracks were selected and optimized on the day of flight (avoiding light, variable, or shifting winds; poorly developed mixed layers; and frontal systems) to minimize analysis errors due to uncertain meteorology. Along with mixed-layer surveying, each flight included 1–2 vertical profiles to characterize the atmosphere’s vertical structure from the surface to lower free troposphere. The GEM flights also included extensive point source characterization as described by Yu et al. (2020).

A cavity ring-down spectrometer (CRDS G2301 for GEM1, G2210-m for GEM2 and GEM3; Picarro Inc., USA) was deployed on the aircraft to quantify methane, ethane (C_2_H_6_, GEM2 and GEM3 only), water vapor (H_2_O) and carbon dioxide (CO_2_) mole fractions at 1Hz. Ground-based calibrations employed compressed ambient-level gas cylinders traceable to National Oceanic and Atmospheric Administration (NOAA) Global Monitoring Laboratory (GML) standards on the WMO X2004A CH_4_ calibration scale. The instrumental precision for methane is <1ppb, and the overall accuracy is estimated at <3.5ppb based on the expanded uncertainties for the calibration standard. We use 1min averaged observations here to constrain regional fluxes. Additional onboard observations included nitrous oxide (N_2_O), carbon monoxide (CO), H_2_O, and CO_2_ mole fractions by continuous-wave tunable infrared laser absorption spectrometry (0.5Hz, Aerodyne Research Inc., USA) as described by Gvakharia et al. (2018); ozone (O_3_) mole fractions (0.2Hz; dual-beam ultraviolet spectrometer, model 205, 2B Technologies Inc., USA); temperature and relative humidity (1Hz; model HMP60, Vaisala Corp., Finland); and GPS location, wind speed and direction, ambient pressure, and other relevant flight parameters as described by Yu et al. (2020).

### 2.2 Forward modeling framework

#### 2.2.1 GEOS-Chem methane simulation and prior emissions

We use the GEOS-Chem CTM (v11–02; http://acmg.seas.harvard.edu/geos, last access: 11 January 2021) and its adjoint (v35) to optimize regional methane emissions. Simulations are performed on a nested 0.25° × 0.3125° grid over North America (9.75–60° N, 60–130° W; Fig. 1) using GEOS-FP meteorological fields from the National Aeronautics and Space Administration (NASA) Global Modeling and Assimilation Office (GMAO, 2013), with 5 and 10min time steps for transport and emissions, respectively. The 3-hourly dynamic boundary conditions (BC) are from global simulations at 2° × 2.5° and bias-corrected as described later. Initial conditions are obtained from a 25-year global spin-up at 2° × 2.5° (bias-corrected in the same manner), followed by a 30d high-resolution (0.25° × 0.3125°) spin-up over our nested domain.

Prior methane emissions in the model are as follows. Wetland emissions use the WetCHARTs ensemble mean (Bloom et al., 2017), uniformly scaled up by 10 % to match the global estimate from Kirschke et al. (2013). Anthropogenic emissions use the GEPA inventory (Maasakkers et al., 2016) over the US (which includes seasonally varying livestock and rice emissions and aseasonal fossil fuel, waste, and industrial emissions). Anthropogenic emissions elsewhere are based on EDGAR v4.3.2 (2017), except Canadian and Mexican oil and gas emissions, which use CanMex (Sheng et al., 2017). Emissions from biomass burning use the Quick Fire Emissions Dataset (QFED) (Darmenov and Silva, 2015), and those from geological seeps and termites follow Maasakkers et al. (2019) and Fung et al. (1991), respectively. Simulations include a set of tagged tracers to track methane from relevant source sectors as detailed in Sect. 2.3.

**Figure 2.**
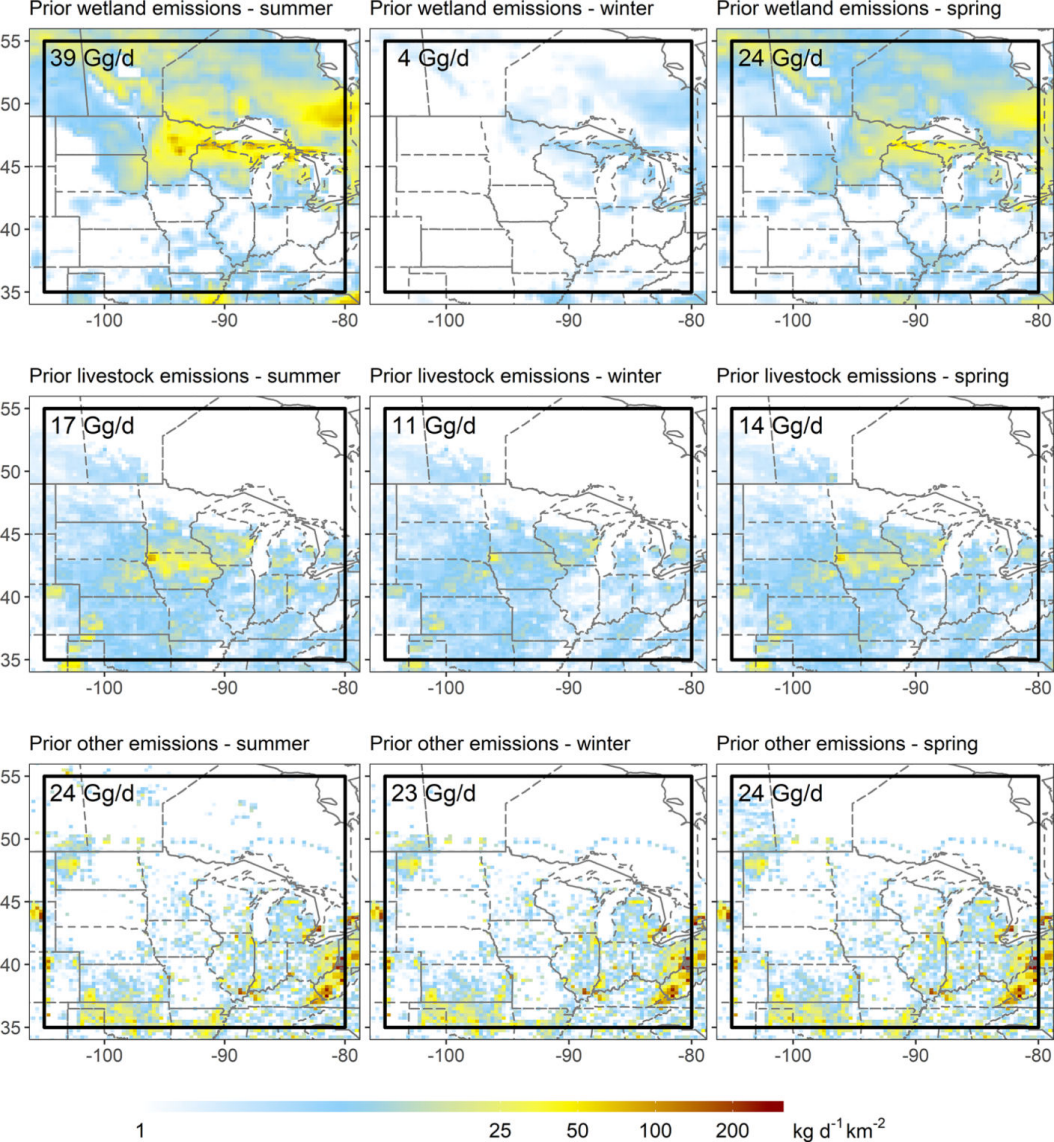
Prior methane emissions in the Upper Midwest for the GEM 1–3 flight periods (GEM1 – summer, 20 July–24 August 2017; GEM2 – winter, 3–28 January 2018; GEM3 – spring, 7 May–2 June 2018). Emission inventories are as described in the text. The black box indicates the inversion domain.

Our analyses focus on the Upper Midwest, defined here to include the north central US and south central Canada region shown in Fig. 1. Figure 2 maps the prior emissions for summer, winter and spring. According to the above inventories, wetlands (36 % of the total annual flux) and livestock (23 %) represent the two largest regional methane sources. Natural gas and petroleum systems, wastewater and landfills, coal mines, and other sources contribute the remaining 15 %, 12 %, 9 %, and 5 %, respectively. Seasonality in the prior emissions is dominated by wetlands; these vary from 39 Gg/d in July–August 2017 (GEM1) to 4 Gg/d in January 2018 (GEM2), with an onset in late May during the GEM3 timeframe. The prior livestock emissions vary from 17Gg/d in July–August 2017 (GEM1) to 11 Gg/d in January 2018 (GEM2) due to the temperature-dependent manure source. Figure 2 shows that wetland emissions are concentrated in the north of the Upper Midwest domain, whereas livestock and other anthropogenic emissions occur predominantly to the south. This spatial separation provides an important advantage for resolving source contributions in our inversions.

The major atmospheric methane sink (90 % of the total loss) is oxidation by hydroxyl radical (OH), computed in the model using archived 3-D monthly OH fields from a full-chemistry simulation (v5–07-08). Other loss processes include stratospheric oxidation (6 % of the total sink), computed using archived monthly loss frequencies from the NASA Global Modeling Initiative (Murray et al., 2013); soil absorption (3 %), computed following Fung et al. (1991); and tropospheric oxidation by chlorine (Cl, 2 %), computed using archived 3-D monthly Cl fields from Sherwen et al. (2016). The resulting global tropospheric methane lifetime in our simulations is 12 years.

#### 2.2.2 Evaluating model boundary and initial conditions

Given the large atmospheric methane burden (1850–1950 ppb) relative to the magnitude of North American enhancements (up to 200 ppb in our prior simulations), careful background evaluation is needed to avoid a biased source optimization. We therefore use measurements over the remote Pacific from the Atmospheric Tomography Mission (ATom; flight tracks shown in Fig. 1) to evaluate and correct the model boundary and initial conditions. ATom featured pole-to-pole sampling with continuous vertical profiling (0.2–12 km) and onboard measurements including methane (Picarro model G2401m, Picarro Inc., USA) and a wide suite of other atmospheric species (Wofsy et al., 2018).

Figure S1 compares tropospheric background methane measurements (represented as 0.1 quantiles within 1° latitude bins) from ATom3 (September–October 2017; flight altitudes ≤ 10 km) and ATom4 (April–May 2018; flight altitudes ≤ 8 km) with GEOS-Chem predictions along the flight tracks. The model–measurement background difference over North American latitudes averages 5.4 ppb (0.3 %) for ATom3 and 9.2ppb (0.5 %) for ATom4. We correct the model boundary and initial conditions using a smoothed spline fit of this 0.1 quantile difference to latitude, with GEM1 (July–August 2017) and GEM2 (January 2018) corrected based on ATom3 and GEM3 (May–June 2018) corrected based on ATom4.

Finally, as described later we assess the potential impact of any residual model background errors through a set of sensitivity inversions in which the bias-corrected boundary conditions are included in the state vector for further optimization. Results are described in Sect. 2.5 and employ a 0.4 % background error standard deviation based on the above model–measurement disparities.

#### 2.2.3 Assessing meteorological uncertainties

We use two approaches to assess the potential impacts of model transport errors on our findings. First, we test whether a misrepresentation of regional-scale synoptic transport could bias our inversion results by evaluating the optimized model against independent datasets from different years, as described in Sect. 2.4. Second, we assess model uncertainties in vertical mixing using planetary boundary layer (PBL) depth estimates derived from balloon-based radiosonde profiles in the Integrated Global Radiosonde Archive Version 2 (IGRA v2). We use 00:00UTC (18:00 or 19:00 local standard time) sonde launch data from six sites in the Upper Midwest (Fig. 1, red triangles) during August 2017 (GEM1), January 2018 (GEM2), and May 2018 (GEM3) in this analysis. Depending on season, the 00:00UTC sounding can occur after the collapse of the daytime mixed layer, but the preceding day’s PBL depth can still generally be determined from vertical temperature and dew point transitions atop the residual layer. The resulting PBL estimates are then compared with the mean midday (12:00–16:00) value in the model. Figure S2 shows that the resulting model PBL biases average less than 10 %, with mean model:measurement ratios of 0.98, 0.97 and 0.90 for summer, winter and spring, respectively. While the GEOS-FP daytime mixing heights were shown previously to be biased high (by 30 %–50 %) over the US Southeast during summer (Millet et al., 2015), we find here that no such bias manifests over the Upper Midwest.

### 2.3 Inverse modeling framework

We quantify methane emissions in the Upper Midwest using a multi-inversion framework that combines (1) sector-based analytical inversions, with the prior spatial distribution of emissions taken as a hard constraint; (2) spatial and sectoral clustering of grid cells using a Gaussian Mixture Model (GMM), with subsequent analytical optimization; and (3) application of the GEOS-Chem adjoint to spatially optimize fluxes on the 0.25°× 0.3125° model grid. The above inversions employ widely differing assumptions and constraints, and together they allow us to identify robust aspects of the derived methane flux fields and quantify the sensitivity of results to these assumptions. We perform the above inversions separately for each season (summer: GEM1; winter: GEM2; spring: GEM3). Inversion performance is discussed in Sect. 2.5.

#### 2.3.1 Cost function and error specification

All inversions in this study optimize methane emissions by minimizing the Bayesian cost function *J* (***x***):

(1) $$J\;(x)={\left(x-x_{a}\right)}^{T}S_{a}^{-1}\left(x-x_{a}\right)+\gamma {\left(y-F(x)\right)}^{T}S_{\text{O}}^{-1}(y-F(x)),$$

where ***x*** is the state vector to be optimized (defined differently for the various inversion frameworks), ***x***_*a*_ is the vector of prior emissions, **S**_*a*_ is the error covariance matrix for the prior emissions, *y* and *F* (***x***) are the observed and simulated methane mixing ratios along the GEM flight tracks, respectively, and **S**_O_ is the error covariance matrix for the observing system (including both measurement and model contributions). The regularization parameter *γ* balances the prior and observational contributions to *J (****x****)* and is set to 10 for our base-case analyses as discussed in the Supplement.

Prior errors are prescribed as follows. Wetland emission uncertainties are based on the standard deviation (*σ*) of the WetCHARTs ensemble on the 0.25° × 0.3125° model grid, averaging 140 % for summer (*σ* = 55 Gg/d) and spring (*σ* = 34 Gg/d) and 310 % for winter (*σ* = 12 Gg/d) on the Upper Midwest domain of Fig. 1. For anthropogenic emissions, we employ a scale-dependent uncertainty (encompassing magnitude and displacement uncertainties) following Maasakkers et al. (2016); the resulting error standard deviation averages 40 %–105 % across sectors over our study region. For other sources we assume a prior error standard deviation of 50 % following earlier studies (Maasakkers et al., 2019; Turner et al., 2015; Wecht et al., 2014; Zhang et al., 2018; Sheng et al., 2018b). For inversions optimizing the total methane flux across sectors, the above terms are combined in quadrature as the diagonal elements of the prior error covariance matrix.

The adjoint 4D-Var inversions derive methane emissions at 0.25° × 0.3125° resolution, and in this case we use a 200 km length scale (decaying exponentially) to populate the off-diagonal elements of the prior error covariance matrix. Previous methane inversions by Wecht et al. (2014) and Monteil et al. (2013) assumed length scales of 275–500 km to further smooth the solution. In our case the analytical inversions impose strict error correlation by spatial cluster or source sector; thus, the adjoint and analytical analyses together span a wide range of error correlation scenarios. Since the analytical inversions solve for emissions by sector or by aggregated region, we employ diagonal prior errors in those cases.

The observational error covariance matrix is constructed from the residual standard deviation of the observation–prior model difference across a 2° × 2° moving window (Heald et al., 2004). The resulting error standard deviation, including forward model and instrumental contributions, averages 26 ppb and is assumed diagonal. The overall observing system error is hence dominated by forward model and representation errors rather than by the *<* 1 ppb measurement precision.

#### 2.3.2 Sector-based inversions

We first derive an optimized set of methane emissions by solving d*J* (***x***)/d***x*** analytically by sector. Seven state vector elements are thus optimized across the nested model domain, representing emissions from (1) wetlands, (2) livestock, (3) fossil fuel, (4) rice, (5) biomass burning, (6) other anthropogenic emissions (landfill, waste water, and other) and (7) other natural emissions (geological seeps and termites). Over the timescale and spatial scale of our inversions the methane emission–concentration relationship is linear, and we thus construct the Jacobian matrix **K** using tagged tracers for each of the above source sectors. The sector-based inversions offer the advantage of direct source attribution but with increased potential for aggregation error given the prescribed emission distributions.

#### 2.3.3 GMM inversions

The GMM inversions cluster individual (∼25km) grid cells with similar emission characteristics, and then analytically optimize methane fluxes by cluster. GMM is a probabilistic approach that assumes each subpopulation (or cluster) is a multivariate Gaussian distribution (i.e., each cluster is ellipsoidal and centered in the feature space) (Turner and Jacob, 2015). We use an expectation-maximization algorithm (Dempster et al., 1977) to find the maximum-likelihood GMM classification for seven emission sectors in the Upper Midwest (wetland, livestock, fossil fuel, rice, biomass burning, other anthropogenic emissions and other natural emissions) and for total emissions in other regions. In each case the number of clusters Γ ∈ [1, 9] is selected based on the Bayesian Information Criterion (Schwarz, 1978), with low-emission clusters (e.g., termites and seeps) grouped to avoid weak sensitivity in the Jacobian matrix. Sector-specific clusters in the Upper Midwest are defined using eight mean- and variance-normalized variables: latitude, longitude, grid-level prior sectoral emissions (three seasons) and grid-level scaling factors (SFs; iteration 8; three seasons) derived from the adjoint 4D-Var inversions. Emission clusters for other regions are defined using the above eight variables (for total emissions) and the prior sectoral emission fractions (seven sectors × three seasons). In this way we identify a total of 28 GMM clusters (Fig. S3), construct the Jacobian matrix **K** based on the associated sensitivities in simulations with tracers tagged to these 28 clusters and solve d*J* (***x***)/d***x*** analytically. The GMM inversions thus derive sector-resolved methane fluxes along with their general spatial distributions. They provide a middle ground between the source-resolved but spatially constrained sector-based inversions above and the spatially resolved but source-agnostic adjoint 4D-Var inversions below.

#### 2.3.4 Adjoint 4D-Var inversions

The adjoint 4D-Var inversions optimize total methane emissions on the 0.25° × 0.3125° model grid *via* iterative minimization of d*J* (***x***)/d***x*** in a quasi-Newtonian routine (Henze et al., 2007). The resulting state vector contains 6400 elements over the Upper Midwest domain (Fig. 1), thus enabling detailed spatial corrections to the prior emissions on a ∼25 km scale. To avoid overfitting, we impose a 200km prior error correlation length scale as described previously. We further perform a suite of sensitivity inversions to evaluate the robustness of the derived emissions by varying the initial scale factors (i.e., employing the GMM-derived scale factors as the initial guess in the adjoint optimization, referred to as GMM-ADJ in the following) and by varying the regulation parameter *γ* ∈ [0.1, 1000] and thereby the weight of the prior versus observational cost function terms. In all cases convergence to the final result is ascertained based on a cost function reduction per iteration < 2.5 % of *J*_0_.

### 2.4 Independent measurements for evaluation

We evaluate our top-down methane emission estimates using the independent airborne and tall tower datasets shown in Fig. 1 and described below. Datasets are calibrated using standards traceable to the WMO X2004A calibration scale, with overall accuracies < 4ppb in all cases (Davis et al., 2018; Andrews et al., 2017; Richardson et al., 2017). Comparisons are based on 5s (aircraft) and 1h (tower) averaged data, with the model sampled at the time and location of measurement.

1. *ACT-America airborne measurements.* The Atmospheric Carbon and Transport-America (ACT-America) campaign (Davis et al., 2018; DiGangi et al., 2017) featured methane measurements from two aircraft platforms, in both cases by CRDS (2401 m, Picarro Inc., USA) at 1Hz frequency (Davis et al., 2018; Baier et al., 2020). We employ within-PBL methane observations from ACT-America flights during July–August 2016, October–November 2017 and April–May 2018 to evaluate GEM inversion results for summer, winter and spring, respectively. The 5s average measurements and along-track model output are both aggregated to the model grid and time step prior to intercomparison. Flights selected for inversion evaluation occurred over and downwind of the Upper Midwest (Fig. 1), mainly sampling the southern portion of our domain. Livestock (29 % of the mean simulated enhancement), fossil fuel (28 %) and wetlands (26 %) are the three largest methane source influences along these flight tracks based on the prior GEOS-Chem tagged tracer simulations.
2. *WSD tall tower measurements.* Methane is measured at WSD (Wessington, South Dakota; 44.05° N, 98.59° W, 592m above sea level (a.s.l.); Miles et al., 2018) by CRDS (CFADS2401 or CFADS2403; Picarro Inc., USA) from a single inlet at 60m above ground level (a.g.l.). The WSD tower is located in the southwest of our analysis region, and thus captures the influence of long-range transport under westerly winds and of Upper Midwest emissions under easterly winds. Based on the prior tagged tracer simulations, wetlands (45 % of the mean simulated enhancement) and livestock (30 %) are the two largest methane source influences at WSD during summer and spring. In winter, livestock (43 %) and fossil fuel (41 %) sources predominate.
3. *KCMP tall tower measurements.* Methane is measured at KCMP (Rosemount, Minnesota; 44.69° N, 93.07° W, 290 ma.s.l.; AMERIFLUX, 2018; Chen et al., 2018) by tunable-diode laser absorption spectroscopy (TGA200A, Campbell Scientific Inc., USA) from two air sampling inlets at 3 and 185 m a.g.l. The KCMP tower is located 25 km south of the Minneapolis–Saint Paul metropolitan area and samples a predominantly agricultural footprint (easterly, southerly and westerly winds), along with urban and wetland influences (northerly winds). The main methane source influences at KCMP according to the prior GEOS-Chem simulations are from wetlands (50 %–56 % of the mean simulated enhancement) and livestock (22 %) during spring and summer and from livestock (39 %) and fossil fuel (27 %) during winter.
4. *LEF tall tower measurements.* Methane is measured at LEF (Park Falls, Wisconsin; 45.95° N, 90.27° W, 470 ma.s.l.; Desai et al., 2015; Andrews et al., 2017) by cavity-enhanced absorption spectroscopy (LGR 9080001 Fast Methane Analyzer, Los Gatos Research, Inc., USA). Measurements are performed sequentially from three air sampling inlets at 30, 122 and 396 m a.g.l. based on the protocol described by Andrews et al. (2014). The LEF tower is located in the northeast of our analysis region within a mixed wetland and forest landscape. LEF features a larger influence from natural emissions than the datasets above: based on our prior simulations, wetlands contribute > 67 % of the mean methane enhancement during summer and spring (versus 44 %–56 % for the other tall towers); livestock contribute an additional 15 %. In winter, fossil fuels (34 %) and livestock (31 %) drive the largest concentration enhancements.

In the case of the tall tower measurements, we use two approaches to evaluate our inversion results. First, we test the optimized model against tall tower data contemporaneous with the GEM flights (August 2017, January 2018, May 2018). Second, we test the optimized model against tall tower data for the same month in a different year (August 2018, January 2017, May 2017). The latter test guards against overfitting to the GEM data; for example, erroneously adjusting emissions to compensate for broadscale model transport errors during the GEM timeframe. In both cases we employ daytime (10:00–18:00 LT) data for model–measurement comparison. The WSD tower was not yet established in January 2017, and thus only the later comparisons are possible here. In all cases we use observations from the highest available inlet, with the model sampled at the corresponding vertical level, to ensure the widest fetch for sampling regional emissions while minimizing near-field influences.

### 2.5 Inversion performance

All inversions lead to a significant reduction in the cost function, with the adjoint 4D-Var and GMM inversions tending to yield larger decreases (36 %–97 %) than the sector-based inversions (12 %–43 %). The adjoint 4D-Var and GMM inversions are able to optimize the spatial distribution of emissions, improving the posterior fit to the data and reducing aggregation error.

**Figure 3.**
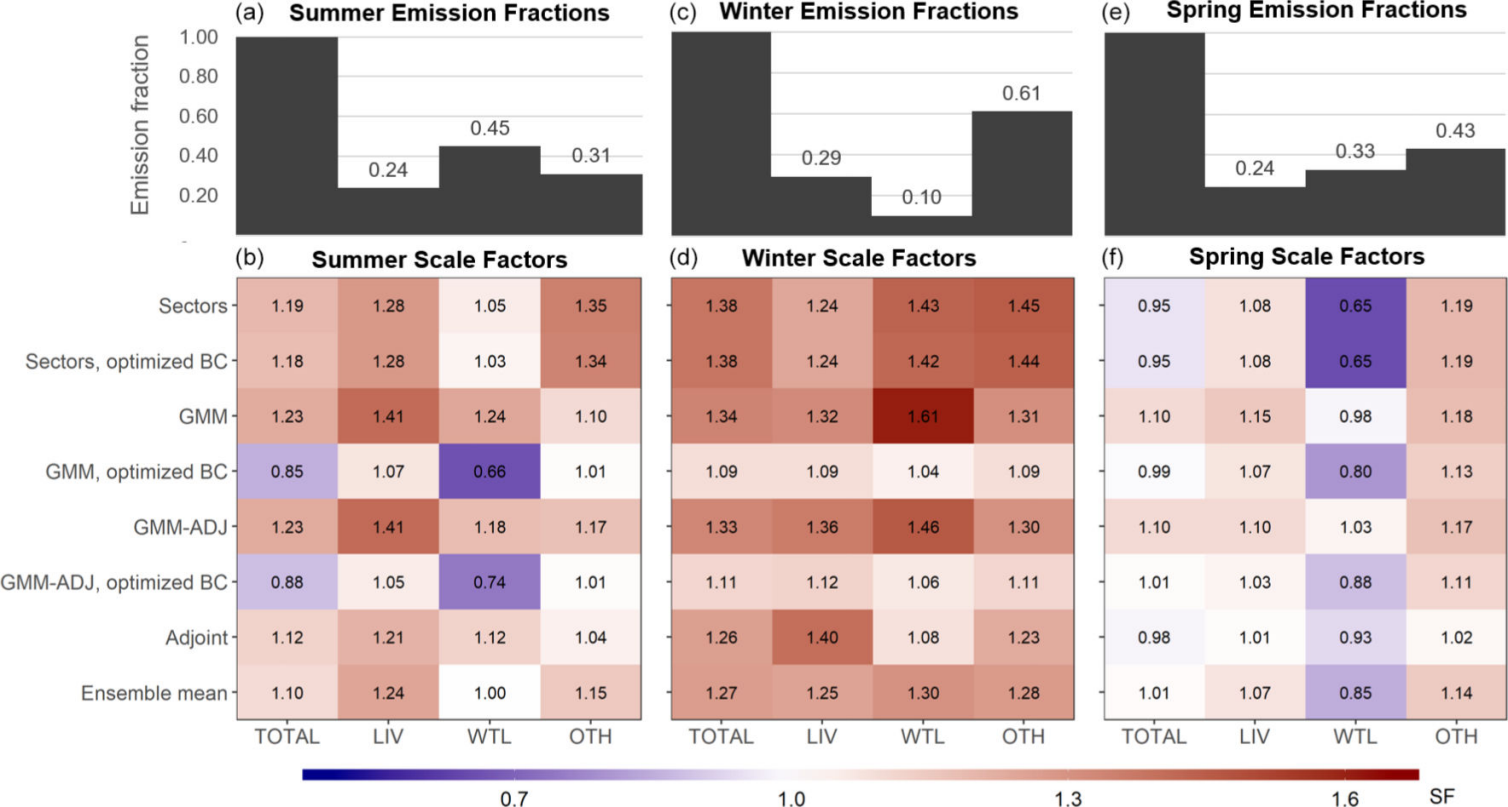
Methane emission scale factors by sector derived from the multi-inversion analysis over the Upper Midwest (black box in Fig. 1). Results are shown for GEM1 (**a, b**; July–August 2017), GEM2 (**c, d**; January 2018) and GEM3 (**e, f**; May–June 2018). Matrix columns show aggregated regional scale factors for total methane emissions (TOTAL), livestock (LIV), wetlands (WTL), and other sources (OTH). Rows show results from seven individual inversions (for details, see Sect. 2.3) along with the ensemble mean. Bar plots in the top row show the emission fractions for each source grouping based on the ensemble-mean inversion results. Boundary condition scale factors for the corresponding sector-based and GMM inversions are 1.00/1.02 (summer), 1.00/1.01 (winter) and 1.00/1.00 (spring), respectively.

Figure 3 shows that the derived adjustments to the total regional methane flux are consistent across inversion frameworks. Specifically, results point to a wintertime emission underestimate and to very modest (< 10 %) springtime corrections. More variable results are obtained during summer; however, even here the derived total flux adjustments are ≤ 23 % in all cases.

The sector-based and GMM inversions enable direct source attribution, and we attribute the adjoint-derived emissions based on the prior grid cell source fractions. We find in this way that (as with the total flux) inversion results are also generally consistent on a sectoral level: uniformly upward adjustments are derived in winter, whereas springtime results point to a wetland overestimate but to only minor corrections for other sources. As before, sectoral results are more variable during summertime; this point is further discussed below. Finally, we show later that geographically consistent emission adjustments are obtained across the set of spatially explicit inversions, further supporting the robustness of our findings.

The largest disparities in Fig. 3 occur when the methane boundary conditions are optimized in the inversion rather than prescribed: total regional emissions derived in this way are ∼15 %–25 % lower than the ensemble mean during summer and winter. The summertime wetland emissions exhibit the strongest such sensitivity, reflecting imperfect seasonal wetland–background separation in the GEM data. In particular, the only downward adjustments (up to 34 %) to the summer wetland flux are derived when optimizing boundary conditions; all other inversions yield ≤ 24 % positive corrections. These same disparities account for the largest spread in derived total flux estimates for summer (scale factors of 0.85 versus 1.23). We show below that inclusion of the boundary conditions in the state vector for optimization does not consistently improve model performance, supporting the prior use of ATom data for this purpose.

We performed a series of sensitivity inversions to test how our results depend on the weighting of the observational versus prior components of the cost function, the prior wetland emissions, and the prior oil and gas emissions. Results are detailed in the Supplement and show that our overall findings are robust across these tests. In the case of the oil and gas sensitivity analysis, we find in particular that (i) our wetland and livestock estimates are not strongly sensitive to fossil fuel-related emission errors and that (ii) the derived oil and gas fluxes are prior-dependent and only weakly constrained by the GEM observing system.

**Figure 4.**
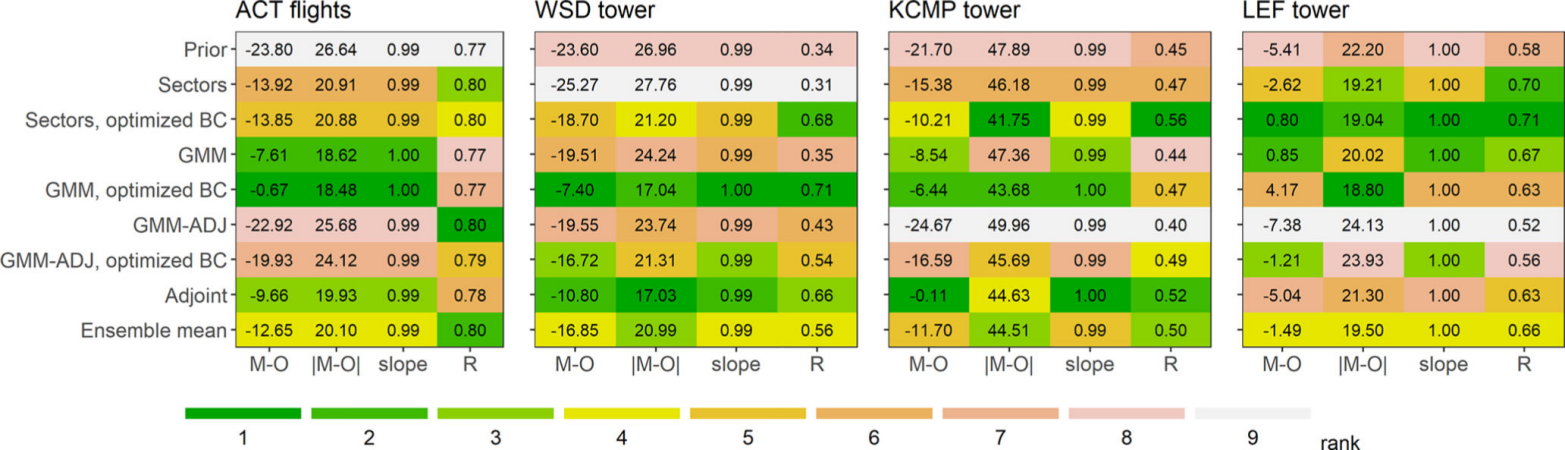
Inversion performance evaluation against independent observations from alternate years. Evaluation datasets include airborne measurements from the ACT-America campaign and tall tower measurements from Wessington South Dakota (WSD), Rosemount Minnesota (KCMP), and Park Falls Wisconsin (LEF). Each matrix displays summary performance statistics for the seven inversions, and for the ensemble mean, with respect to the indicated evaluation dataset. Columns in each matrix show the model mean bias (M-O; ppb), mean absolute bias (|M-O|; ppb), model:measurement slope (note this is within 1 % of unity in all cases) and model:measurement Pearson’s correlation coefficient (*R*). Values are colored by rank for the above criteria. See Sect. 2.4 for details.

In nearly every case, the simulations with optimized emissions agree more closely with independent aircraft and tall tower measurements than the prior simulations do (Fig. 4). Exceptions include (i) the sector-based inversion versus the WSD tower data and the GMM-ADJ inversion versus the KCMP and LEF tower data. The former likely reflects aggregation error in the spatially constrained sectoral optimization. The latter suggests overfitting: the GMM-ADJ emission adjustments improve model performance during the GEM timeframe (Fig. S4) but not for alternate years (Fig. 4). For all other inversions, the optimized emissions yield performance improvements regardless of the evaluation year, providing a strong argument for the representativeness of the GEM data and the reliability of our emission adjustments.

Together, the ensemble of inversions provides an envelope of solutions for assessing the robustness and uncertainty of the results. Below, we discuss emergent findings that are consistent across inversions and diagnose the associated level of confidence based on the spread in results.

## 3 Optimized methane emissions in the Upper Midwest

Averaging our seasonal inversion results with the prior values for fall, we find that wetlands represent the single largest (32 [29–35] %) methane source in the Upper Midwest at 20 [16–23] Gg/d. Here and below, reported central values and uncertainties reflect the mean and range across our inversion ensemble. Anthropogenic sources collectively account for the remaining 68 [65–71] %, with livestock making the largest individual contribution (15 [14–17] Gg/d). Smaller but still significant sources are derived for natural gas and petroleum systems (10 [9–11] Gg/d), waste and landfills (8 [7–8] Gg/d), and coal mines (6 [5–7] Gg/d); however, as noted above these latter estimates are strongly influenced by the prior. Given the predominant role for livestock and wetlands, we focus on these sources and proceed to discuss the above findings in detail by season.

### 3.1 Summer (GEM1): spatial errors in the prior wetland flux and an underestimate for livestock

Figure 3 shows that the GEM aircraft data broadly support the total prior summertime methane emissions for the Upper Midwest, with a derived correction factor of 1.10 [0.85–1.23]. The resulting posterior seasonal flux is 88 [68–99] Gg/d. On a sectoral basis, wetlands provide the dominant seasonal emission source (45 %, 39 [26–49] Gg/d). Livestock account for 24 % (21 [18–24] Gg/d), with the remaining 31 % (27 [24–32] Gg/d) including a derived 16 [14–21] Gg/d from fossil fuels (including coal mines) and 8 [8–9] Gg/d from wastewater.

**Figure 5.**
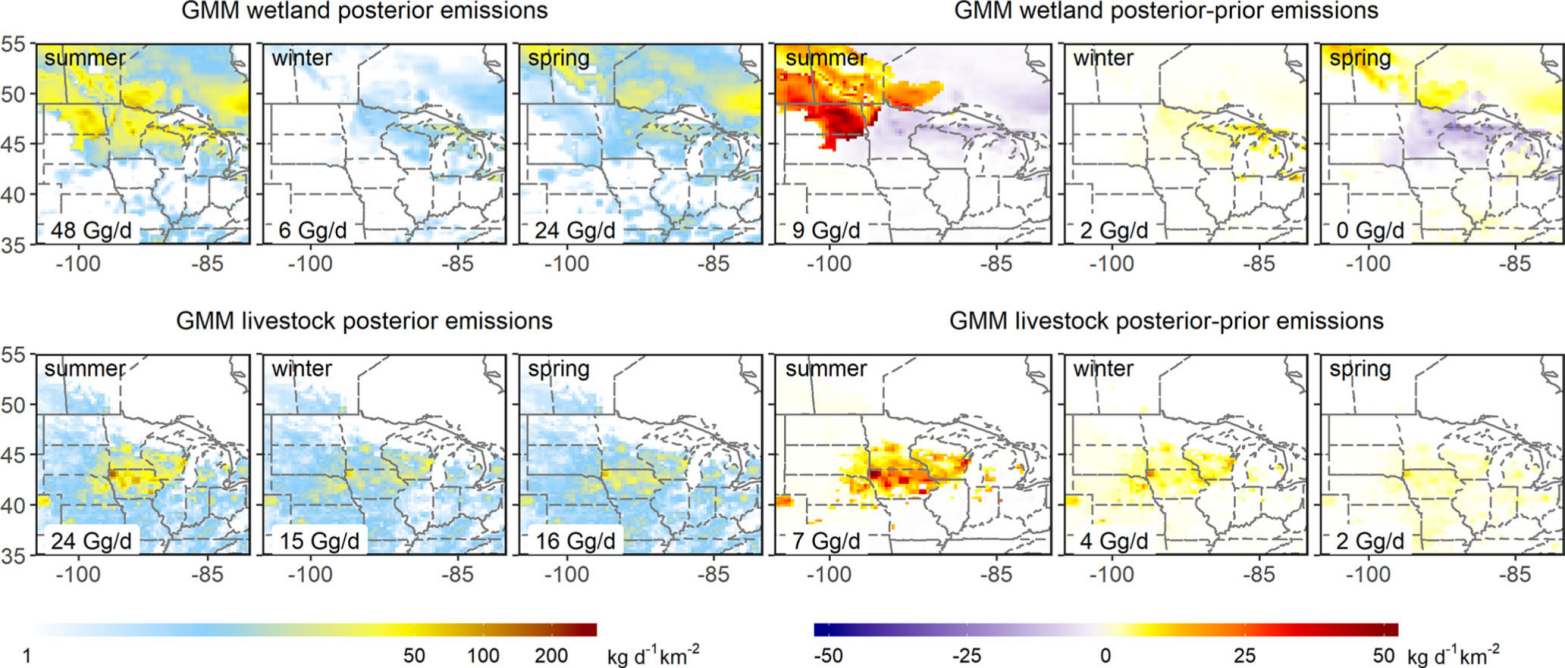
Wetland and livestock methane emissions derived from the GMM inversions with associated posterior–prior differences. Results are shown for GEM1 (July–August 2017), GEM2 (January 2018) and GEM3 (May–June 2018).

**Figure 6.**
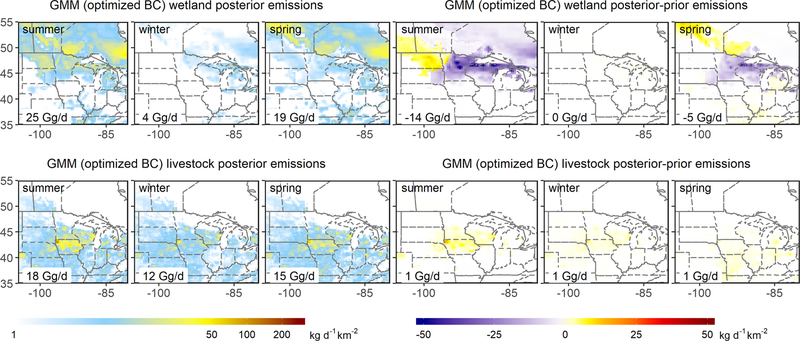
The same as Fig. 5 but showing results for the GMM inversions with boundary condition optimization.

**Figure 7.**
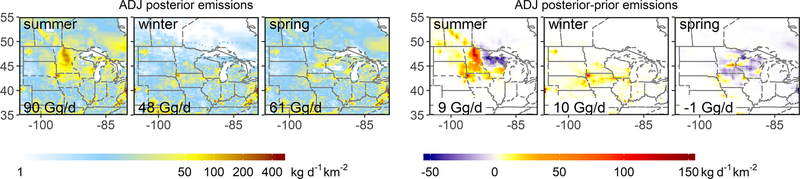
Methane emissions derived from the adjoint 4D-Var inversions with associated posterior–prior differences. Results are shown for GEM1 (July–August 2017), GEM2 (January 2018) and GEM3 (May–June 2018). GMM-ADJ results are similar (Figs. S7–S8).

While the optimized summertime wetland fluxes agree reasonably well with the WetCHARTs estimate for the region as a whole (mean scale factor of 1.00 [0.66–1.24]), this is fortuitous: the inversions point to significant (offsetting) spatial errors in the prior. Figures 5–7 show that the individual inversions reveal a wetland underestimate in the northwest of our domain (reaching 76mg/m^2^/d) but an overestimate in the northeast (reaching −77mg/m^2^/d). These spatial patterns are robust across the inversions, but the adjustment magnitudes differ – for example, the GMM inversion yields much stronger upward adjustments in the northwest (Figs. 5–7). We attribute this spread in part to the imperfect wetland–background separation discussed earlier.

The northwest wetlands lie predominantly in the Prairie Pothole region of the eastern Dakotas and Canada and have highly variable hydrology driven by snowmelt, precipitation, and groundwater inflow. Based on 1997–2009 data, these wetlands have been declining at a rate of ∼25km^2^/yr (USF&WS National Wetlands Inventory, 2019). Areas to the northeast mainly feature coastal wetlands under the influence of the Great Lakes, which based on 2004–2009 data have undergone recent expansion by 11 km^2^/yr (USF&WS National Wetlands Inventory, 2019). Our findings here suggest that methane emissions from Great Lake coastal wetlands (while increasing over time) are presently overestimated, while prairie pothole emissions (while decreasing over time) are presently underestimated.

We further infer from the GEM aircraft measurements a summertime underestimate in regional anthropogenic emissions (Fig. 3). In particular, the GEPA prior livestock emissions increase by 24 % (4 Gg/d) in the multi-inversion average, with scale factors ranging from 1.05–1.41 (1–7 Gg/d). As seen earlier for wetlands, the lowest scale factors (1.05, 1.07) are obtained when the boundary conditions are allowed to vary in the optimization, with other inversions pointing to a 21 %–41 % (4–7 Gg/d) livestock flux underestimate. The individual inversions are spatially consistent in showing the livestock underestimate manifesting most strongly in the center of the Upper Midwest domain (Iowa/southern Minnesota/southern Wisconsin; Figs. 5–7). Anthropogenic emissions other than livestock are adjusted upward through the inversions by 15 [1–35] % (4 [0–8] Gg/d) in a relatively consistent manner across the region (Figs. 5–7).

### 3.2 Winter (GEM2): an emission underestimate across sectors

All inversions indicate that wintertime methane emissions are underestimated in the prior inventories, with an ensemble-mean scale factor for the total regional flux of 1.27 [1.09–1.38]. We thus obtain a seasonal methane flux of 49 [42–53] Gg/d that is dominated by anthropogenic emissions from fossil fuel (37 %, 18 [15–20] Gg/d), livestock (29 %, 14 [12–16] Gg/d), and wastewater (20 %, 10 [8–11] Gg/d). Regional wetland emissions are minor (10 %, 5 [4–6] Gg/d) during winter, and we therefore focus the following discussion on anthropogenic sources.

We find that wintertime livestock emissions (enteric fermentation and manure management) are underestimated by 25 % (3 [1–5] Gg/d) in the GEPA inventory and that this disparity is most pronounced over the center of the Upper Midwest (Iowa/southern Minnesota/southern Wisconsin). Figures 5–7 show that this is the same area where we infer a summertime livestock emission underestimate of comparable magnitude (24 %, 4 Gg/d). In Sect. 4, we examine the role of enteric fermentation versus manure management in driving these differences.

The wintertime optimization results further point to a 28 [9–45] % (6 [2–10] Gg/d) underestimate of non-livestock anthropogenic emissions, with the largest derived adjustments in the southeast of our domain where fossil fuel sources predominate (Figs. 5–7). Sustained high methane observations during a GEM2 flight over Iowa under southerly winds (Fig. 1) – with up to 100 ppb model–measurement mismatches and co-occurring ethane enhancements – similarly suggest an underestimate of fossil fuel sources to the south of the Upper Midwest, as also diagnosed by Barkley et al. (2019). However, for the purpose of analyses here, we note that a sensitivity inversion omitting this flight does not significantly alter our results.

### 3.3 Spring (GEM3): biased seasonal onset of wetland emissions

The GEM aircraft data indicate that the prior regional flux during springtime is unbiased when taken as a whole: Fig. 3 shows that the ensemble mean correction factor is 1.01 with a range across inversions of 0.95–1.10, resulting in a spring flux of 63 [59–68] Gg/d. On a sectoral basis, wetlands are the largest emission source (33 %, 21 [16–25] Gg/d), followed by livestock (24 %, 15 [14–16] Gg/d), with the remainder including derived contributions of 16 [14–17] Gg/d from fossil fuel and 9 [8–9] Gg/d from wastewater.

While the GEM inversions support the prior springtime methane fluxes in terms of total regional magnitude, results point to biases in the bottom-up wetland emissions and their spatial distribution. Figures 5–7 show that the prior wetland emissions during spring exhibit spatial errors similar to those in summer, with an underestimate to the northwest (reaching 15 mg/m^2^/d) but an overestimate around the Great Lakes (reaching −48 mg/m^2^/d). These spatial errors have smaller peak magnitude (< 63 %) than during summer and lead to a net 15 % wetland flux overestimate for the region as a whole (4 [−1–8] Gg/d; Fig. 3). Upper Midwest wetland methane fluxes in the WetCHARTs inventory used here as prior generally exhibit a sharp onset during late May driven by increasing surface skin temperature (Bloom et al., 2017). The GEM3 flights were conducted during 21 May–2 June 2018 and reveal fluxes that are lower than these predictions. As discussed in the Sect. 4, this implies a bottom-up bias in the timing of the springtime emission onset.

We derive springtime livestock emissions within 7 [1–15] % of the prior estimates (Fig. 3) based on the GEM aircraft measurements. The fractional livestock underestimate in GEPA during spring is thus only 30 % of the summer and winter biases. Since emissions from enteric fermentation – unlike those from manure – have little seasonal dependence (IPCC, 2006), the differing bottom-up biases for summer and winter versus spring point to errors associated with manure management activities; this point is discussed further in Sect. 4.

## 4 Key uncertainties for regional wetland and livestock emissions

### 4.1 Wetland methane fluxes: role of wetland extent and emission temperature dependence

As shown above, the GEM inversions reveal spatial and temporal errors in the WetCHARTs (ensemble mean) prior wetland emissions for the Upper Midwest. Below, we combine the inversion results with the individual WetCHARTs estimates to derive information on key process parameters driving uncertainty in the predicted fluxes.

The WetCHARTs extended ensemble includes 18 members that estimate wetland emissions *F (****t*, *d****)* at time ***t*** and location ***d*** as follows:

(2) $$F\;(t,d)=s\;A\;(t,d)\;R(t,d)q_{10}^{\frac{T(t,d)}{10}}.$$

Here, *A* (***t***, ***d***) is wetland extent (m^2^ wetland area/m^2^ surface area) based on either GLOBCOVER (Bontemps et al., 2011) or the Global Lakes and Wetlands Database (GLWD) (Lehner and Döll, 2004), with temporal variability prescribed using satellite-based surface water or reanalysis-based precipitation datasets (Bloom et al., 2017); *R* (***t***, ***d***) is heterotrophic respiration rate (mgC/d/m^2^ of wetland area) taken as the median monthly value from the Carbon Data Model Framework (CARDAMOM; Bloom et al., 2016); *T* is surface skin temperature (°C); *q*_10_ quantifies the *T* dependence of methane emissions relative to heterotrophic C respiration (i.e. the CH_4_ :C temperature dependence), with *q*_10_ =1, 2, or 3; and *s* is a scaling factor imposing a global flux of 124.5, 166, or 207.5 Tg CH_4_/yr (Saunois et al., 2016; Bloom et al., 2017).

**Figure 8.**
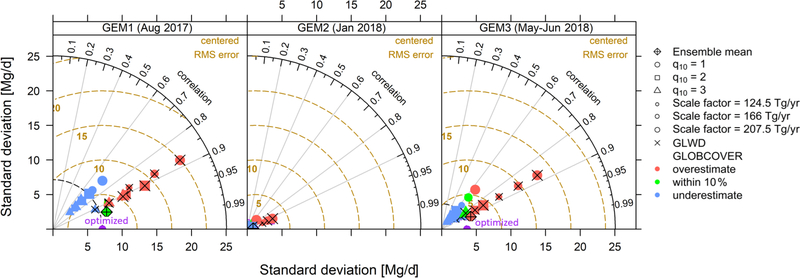
Taylor diagram evaluating the performance of Upper Midwest wetland emission estimates from the WetCHARTs inventory against the optimized fluxes derived here. The colored symbols show the 18 WetCHARTs extended ensemble members, which feature three temperature sensitivity factors (CH_4_ :C *q*_10_ =1, 2, or 3); three scale factors to obtain global emissions of 124.5, 166, or 207.5 Tg/yr; and two wetland extent datasets (GLOBCOVER and GLWD, marked with open symbols and crosses, respectively). Symbols are colored by flux magnitude relative to the optimized emissions. Three statistics are shown in these plots: (1) the slope between each symbol and the origin reflects the spatial correlation between that model and the optimized emissions; (2) the distance between each symbol and the origin reflects the standard deviation of that model estimate; and (3) the distance between each symbol and the optimized value reflects the centered root-mean-square error of that model estimate relative to the optimized solution. Optimized results correspond to the multi-inversion ensemble mean.

Figure 8 shows the agreement between each of the WetCHARTs ensemble members and the optimized wetland fluxes (multi-inversion average) in a Taylor Diagram. It is apparent from Fig. 8 that (1) wetland extent and (2) CH_4_ :C emission temperature dependence (*q*_10_) are major factors controlling prediction accuracy, as discussed further below.

1. *Wetland extent.* We see from Fig. 8 that the GLWD-based models overestimate the actual wetland emissions derived here. However, they also exhibit higher spatial correlation with the optimized fluxes than the GLOBCOVER-based models do. Despite their associated overestimate (also found over the US Southeast; Sheng et al., 2018b), GLWD thus more accurately represents the wetland spatial distribution across the Upper Midwest landscape. The GLWD employs maximum wetland extent estimates derived from a range of sources published during 1992–2000 (DMA, 1992; UNEP-WCMC, 1993; Lehner and Döll, 2004), while the GLOBCOVER data employs year 2009 space-based measurements from Envisat’s Medium Resolution Imaging Spectrometer (Bontemps et al., 2011). However, the mean 2.6-fold difference between the GLWD- and GLOBCOVER-based methane emissions for the Upper Midwest is much greater than any wetland area changes during 2000–2009 (USF&WS National Wetlands Inventory, 2019). This high sensitivity of emissions to wetland extent was likewise demonstrated on a global basis in the WETCHIMP model intercomparison, which reported annual flux estimates varying by ±40 % from the mean with extensive spatiotemporal disparities (Melton et al., 2013).
2. *Temperature dependence* (*q*_10_). We find for both GLWD and GLOBCOVER that a CH_4_ :C *q*_10_ of 3 yields the lowest centered root-mean-square error (RMSE) compared to the optimized fluxes. This corresponds to an average CH_4_ :*Tq*_10_ (i.e., net *T* -dependence for methane emissions) of 5 across the Upper Midwest domain (Fig. S5) versus the prior value of 2.4. Eddy covariance measurements at the Bog Lake peatland site in northern Minnesota (see Fig. 1) during 2015–2017 imply a CH_4_ :*Tq*_10_ of 2.9 but based on 10cm soil temperatures (Deventer et al., 2019). For comparison, Sheng et al. (2018b) found that WetCHARTs ensemble members employing CH_4_ :C *q*_10_ = 1 exhibited the closest agreement with observations for wetlands in the US Southeast.
  However, the bottom-up approach of prescribing *q*_10_ values has inherent limitations, and greater accuracy will require more explicit treatment of underlying drivers. Methane in wetlands is generated through anaerobic microbial metabolism in waterlogged soil, but a separate population of methanotrophic bacteria above the anoxic–oxic boundary can oxidize 50 % or more of that methane before it is able to escape to the atmosphere (Segarra et al., 2015). These competing processes at different depths lead to large uncertainties when defining a single *q*_10_ value – even for an individual site. For example, long-term measurements at the Bog Lake peatland site referenced above reveal large year-to-year CH_4_ :*T* variability associated with water table fluctuations (Feng et al., 2020). Previous site-level studies likewise report a wide range of CH_4_ :*Tq*_10_ values (2–12) depending on location, year and soil temperature depth (Kim et al., 1998; Jackowicz-Korczyński et al., 2010; Mikhaylov et al., 2015; Marushchak et al., 2016; Rinne et al., 2018).

Finally, as discussed earlier, the GEM inversions indicate a bottom-up wetland flux overestimate during spring that may reflect incorrect seasonal timing for the onset of emissions. The Bog Lake peatland eddy covariance measurements support this idea, showing that in many years emissions rise later in the spring than is predicted by the WetCHARTs ensemble mean (Fig. S6). Soil temperatures at depths relevant to microbial processes can exhibit a significant lag relative to the surface skin temperatures used by WetCHARTs for emission estimation (Pickett-Heaps et al., 2011), and we hypothesize that this lag is the primary reason for the springtime discrepancy found here. Such lags vary with environmental conditions such as snow cover, water table, and other factors (Pickett-Heaps et al., 2011). Better characterization of the coupled effects of soil temperature and hydrology on emissions is thus needed to improve the fidelity of methane flux estimates.

### 4.2 Livestock methane: enteric emissions well-represented but large uncertainties for manure

The GEM inversions point to mean underestimates in the prior GEPA livestock emissions of 24 (5–41) %, 25 (9–40) % and 7 (1–15) % in summer, winter and spring, respectively. Below, we explore these discrepancies by partitioning the derived livestock emissions according to the geographic distribution of beef cattle, dairy cattle and hogs.

**Figure 9.**
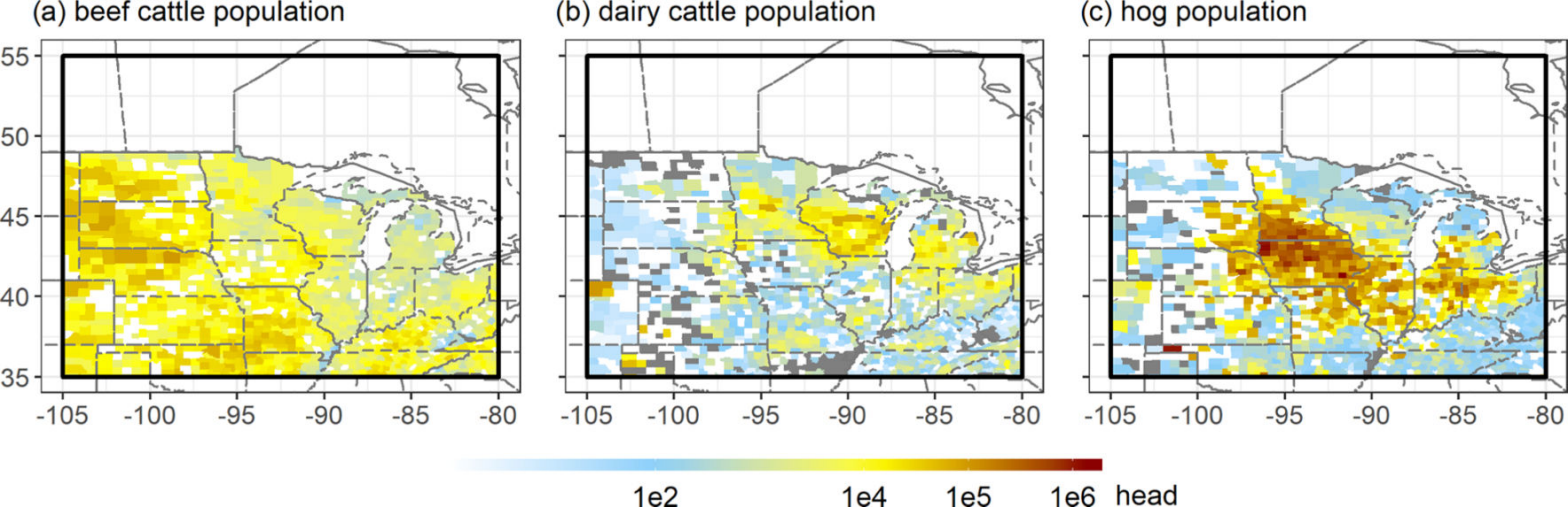
County-level animal populations for (**a**) beef cattle, (**b**) dairy cattle and (**c**) hogs, based on the 2017 US Department of Agriculture Census of Agriculture (USDA-NASS, 2018).

Figure 9 shows county-level animal distributions from the 2017 US Department of Agriculture Census of Agriculture (USDA-NASS, 2018). Beef cattle, dairy cattle, and hogs have distinct spatial distributions, with highest population densities in the Dakotas, Wisconsin and central Minnesota, and Iowa and southern Minnesota, respectively. They also employ different manure management strategies: in our study region, liquid systems, which have > 8× higher methane conversion factors than dry systems (US Environmental Protection Agency, 2016), account for an estimated 1 %, 57 % and 95 % of beef, dairy and hog management activities, respectively. Dry systems make up the remainder. As a result, enteric emissions are thought to account for more than 95 % of the methane flux from beef facilities but only 60 % for dairies (they are minor for hog facilities) (Yu et al., 2020).

The above spatial segregation affords an opportunity to better understand methane emissions by livestock type and (by extension) enteric versus manure contributions. To that end, we partition our optimized fluxes by computing mean livestock emission scaling factors (SFs) separately for model grid cells with beef cattle, dairy cattle or hogs representing ≥ 70 % of the total animal population. Results are shown in Table 1 and reflect statistical averages over 2374 (beef), 260 (dairy) and 1554 (hog) model grids. In each case we present base-case estimates and uncertainties based on the multi-inversion mean and range, respectively.

We find in this way that beef emissions are well-represented in the bottom-up inventory across seasons (base-case adjustments < 15 %; Table 1). On the other hand, the bottom-up dairy cattle and hog emissions exhibit seasonally dependent errors, with a base-case underestimate of ∼30 % in summer and winter but no apparent bias in spring. Taken together, these findings suggest an accurate treatment of enteric emissions in the GEPA inventory but an underestimate of manure emissions with inaccurate seasonality.

The variability of manure emission factors across management systems and their high sensitivity to environmental factors may contribute to the above discrepancies. Along with the large differences between liquid and dry systems, temperature plays a major role in regulating manure emissions, and model misrepresentation of this effect (which can occur, for example, when using surface skin temperatures to approximate manure lagoon temperatures, as in the GEPA inventory) can lead to significant bottom-up errors in both the magnitude and seasonality of predicted fluxes (Park et al., 2006). Local factors such as solar absorptivity, wind, manure depth, pH and humidity can also influence emissions (Rennie et al., 2017; VanderZaag et al., 2013) but are not generally accounted for in inventories. Further, use of lagoon covers and anaerobic digestion systems can reduce methane emissions by up to 90 %, and inadequate information on such factors will lead to inventory errors.

Seasonal manure application is also likely contributing to the bottom-up errors found here. The GEPA inventory computes manure emissions assuming constant on-site manure volume, with seasonal differences arising solely from the temperature dependence of microbial activity (Maasakkers et al., 2016). However, in the Upper Midwest, manure is applied to fields once or more per year, most commonly in spring (MPCA, 2020). This causes manure volume on site to vary significantly by season. Manure emissions after field application are less than 1 % of those occurring during storage and arise mainly from manure-dissolved methane that escapes immediately after application (Amon et al., 2006; Niles and Wiltshire, 2019). We speculate that this factor is the reason the GEM data point to an inventory underestimate for manure in summer and winter but not spring. Inclusion of location-specific information on the timing and rate of manure field application is thus likely to improve bottom-up methane emission estimates.

Additional research will be needed to confirm the role of manure in driving the top-down and bottom-up livestock discrepancies observed here and to pinpoint the primary mechanisms involved. However, our conclusions above are consistent with the domain-aggregated results discussed earlier (Sect. 3) and shown in Fig. 3, as well as with findings from previous studies. For example, a recent bottom-up study using updated animal, feed and management information recommended revising the IPCC 2006 emission factors by +8 % for enteric emissions and +37 % for manure emissions (Wolf et al., 2017). This aligns with our findings here of a +15 % adjustment for beef facilities (dominated by enteric emissions) and an approximate +30 % summer and winter adjustment for dairies and hog facilities (with a larger role for manure emissions). In our previous work, we applied aircraft-based mass balance to quantify facility-level emissions for concentrated animal feeding operations in the Upper Midwest and found (as here) good top-down and bottom-up agreement for enteric emissions but discrepancies for manure (Yu et al., 2020). A recent site-level study at a large Wisconsin dairy farm observed low manure emissions (∼30 % of the enteric flux) owing to frequent field application throughout the year (Wiesner et al., 2020), further supporting our characterization of manure management as a key uncertainty in current large-scale bottom-up inventories.

## 5 Summary and outlook

We applied aircraft measurements from the GEM campaign in a multi-inversion framework to improve understanding of seasonal methane emissions in the Upper Midwest. Together, our optimized emissions for summer, winter and spring (combined with prior results for fall) indicate that wetland emissions account for 32 % (20 [16–23] Gg/d) of the total regional flux during these seasons. Anthropogenic sources make up the remainder, with the largest contribution from livestock (15 [14–17] Gg/d). Smaller but still significant sources are derived for natural gas and petroleum systems (10 [9–11] Gg/d), waste and landfills (8 [7–8] Gg/d), and coal mines (6 [5–7] Gg/d); however, these are only weakly constrained in the inversions by the GEM observations.

Our inversions point to important spatial errors in the WetCHARTs ensemble-mean wetland emissions, with an underestimate in the Prairie Pothole region (reaching 76mg/m^2^/d in summer) but an overestimate for Great Lakes coastal wetlands (reaching −77mg/m^2^/d in summer), and a possible timing bias for the spring emission onset. Based on the WetCHARTs ensemble, wetland extent and emission temperature dependence are the largest uncertainty sources in bottom-up estimates for this region. WetCHARTs estimates based on the GLWD extent dataset tend to overestimate emissions but have higher spatial correlation with the optimized fluxes than GLOBCOVER-based estimates. WetCHARTs estimates employing a CH_4_ :C *q*_10_ of 3 have the lowest RMSE with respect to our posterior emissions, in contrast to findings for the US Southeast where a value of 1 yielded the best model–measurement agreement (Sheng et al., 2018b). However, a body of literature shows that the temperature dependence for methane emissions is highly variable across locations, time and soil depth. Accurate flux predictions will thus require more explicit treatment of underlying drivers including snow cover, water table and the coupled effects of soil temperature and hydrology on emissions.

The optimized livestock methane emissions derived here for the region are ∼25 % higher than the GEPA estimates during summer and winter but agree with the bottom-up estimates (to within 10 %) during spring. Since enteric emissions (unlike those from manure) are relatively consistent throughout the year, this seasonal discrepancy suggests bottom-up errors associated with manure. We propose that the lower emission adjustment during spring reflects management factors such as widespread application of manure to fields at that time.

We further partition the derived livestock emissions based on county-level animal populations for beef cattle (> 95 % enteric emissions), dairy cattle (∼60/40 % enteric/manure emissions) and hogs (mostly manure emissions). In this way we find that enteric fermentation emissions are well-captured by the GEPA inventory with low overall bias but that manure emissions are underestimated by as much as 30 % in summer and winter, with biased seasonality. While further research is needed to confirm this inferred role for manure in driving inventory errors, conclusions here are consistent with other recent work (e.g., Wolf et al., 2017; Yu et al., 2020; Wiesner et al., 2020). Better representation of manure management (for example, accounting for the timing and rate of field application and incorporating finely resolved information on management systems) thus appear to be important priorities for improving bottom-up emission estimates.

Findings here highlight the importance of Upper Midwest agricultural emissions for both the regional (36 % of annual Upper Midwest anthropogenic emissions) and national (∼35 % of North American livestock emissions) methane budget. These emissions should thus receive high priority for mitigation efforts.

Enteric emissions can be reduced through approaches including diet modification, vaccination, nutritional supplements or animal selection; the effectiveness of such approaches and their economic benefits are the subject of a large body of work (Martin et al., 2008; Boadi et al., 2004; Smith et al., 2008; Montes et al., 2013; Nisbet et al., 2020; Hristov et al., 2015). Nutritional changes to reduce methane emissions can influence animal health and decrease plant-available N in fertilizer; additional management is needed to address those issues (Baker et al., 1975; Montes et al., 2013). Enteric emissions can also be reduced by up to 85 % through use of bio-filtration systems; however, in some cases this can lead to increased N_2_O emissions, and assessment of the full facility-level greenhouse gas impact is therefore necessary (Montes et al., 2013; Nisbet et al., 2020).

Manure methane emissions can be reduced by up to 90 % during storage through approaches that suppress methano-genesis (e.g., via manure acidification, slurry aeration or lowered storage temperatures) or capture methane for use (via anaerobic digestion) (Chadwick et al., 2011; Montes et al., 2013). In addition, frequent field application can reduce methane storage time, leading to reduced emissions. However, consideration of crop demand, soil properties and manure nutrient content is needed to avoid exacerbating water pollution and N_2_O emissions (Sutton et al., 2001; Chadwick et al., 2011; Reay et al., 2012).

In the US, 255 anaerobic digestor installations reduced greenhouse gas emissions by 43.8 million metric tonnes CO_2 eq._ while generating 11 billion kilowatt-hours of electricity during 2000–2019 (AgSTAR, 2019). Furthermore, based on the source estimates derived here, application of anaerobic digestion (at assumed 60 % efficiency; Montes et al., 2013) and bio-filtration (85 %; Montes et al., 2013) to all Upper Midwest manure and enteric emissions could in theory yield a 4.5 Tg/yr methane source reduction. This is an upper limit as it assumes uniformly high-efficiency systems and feasibility across all facilities. However, 4.5 Tg/yr is 1.7× the estimated methane flux for the entire Permian basin during 2018–2019 (representing the largest emission ever reported from a US oil- and gas-producing region; Montes et al., 2013; Zhang et al., 2020). Techniques and policies to advance the above management strategies thus have significant potential for methane source mitigation and energy production in the Upper Midwest and nationally.

## Supplementary Material

SI

## Figures and Tables

**Table 1.** Derived methane emission scale factors for livestock by animal category.

Animal category	Analysis counties	Model grid cells	Seasonal scale factors*		
			Summer	Winter	Spring
Beef	646	2374	1.12 [0.98, 1.28]	1.15 [1.05, 1.24]	1.09 [1.04, 1.13]
Dairy	52	260	1.28 [0.97, 1.75]	1.30 [1.14, 1.59]	1.00 [0.81, 1.18]
Hog	428	1554	1.29 [1.09, 1.57]	1.28 [1.12, 1.48]	1.08 [0.93, 1.20]

* Results shown reflect the ensemble mean and range across all inversions and are computed for model grid cells in which the corresponding animal category represents ≥70 % of the total livestock population.

## Data Availability

The GEM aircraft dataset presented in this paper is publicly available online (https://doi.org/10.13020/f50r-zh70, Millet and Yu, 2019).
