# Supplementary material for: Aircraft-based inversions quantify the importance of wetlands and livestock for Upper Midwest methane emissions
Source: Atmos Chem Phys. Author manuscript; Available in PMC 2021 Feb 19. (PMC7894053; doi:10.5194/acp-21-951-2021)
Supplement: SI [file NIHMS1669574-supplement-SI.pdf]

Supplement of Atmos. Chem. Phys., 21, 951–971, 2021
https://doi.org/10.5194/acp-21-951-2021-supplement
© Author(s) 2021. This work is distributed under
the Creative Commons Attribution 4.0 License.

Atmospheric Chemistry and Physics
Open Access

Supplement of

Aircraft-based inversions quantify the importance of wetlands and livestock for Upper Midwest methane emissions

Xueying Yu et al.

Correspondence to: Dylan B. Millet (dbm@umn.edu)

The copyright of individual parts of the supplement might differ from the CC BY 4.0 License.

S1 Additional sensitivity analyses

In addition to examining results across inversion frameworks, we carried out a suite of sensitivity analyses to test how results depend on the weighting of the observational versus prior components of the cost function, the prior wetland emissions, and the prior oil + gas emissions.

First, we performed a series of sensitivity inversions varying the regulation parameter $\gamma \in [0.1, 1000]$ and thereby the relative importance of model-measurement mismatches versus prior departures in the cost function (Eq. 1). We find that our overall results are robust across these tests, with consistent adjustments in terms of their signs and source attribution (Fig. 3 and S9 compare results for γ = 10 and 1). Adjustment magnitudes increase with larger γ due to increased weighting of the observational cost function term. We employ γ = 10 for our base-case analyses as it yields the best overall performance against independent measurements (Fig. S10).

Second, we tested the degree to which our findings depend on the prior wetland emissions employed in the inversions. To this end, we repeated the base-case adjoint optimization using an individual WetCHARTs member (with 137 Tg CH_4/yr globally, CH_4:C q10 = 1, GLOBCOVER wetland extent) as wetland prior, rather than the ensemble mean. This leads to prior wetland emissions that are 29% (spring) and 46% (summer) lower for our study region as a whole compared to the ensemble mean case, with prior differences in specific locations ranging from -70% to +40% (interquartile range) in spring and from -78% to +10% in summer (Fig. S11). When repeating the adjoint inversion in this way, we find that our core conclusions remain robust. Specifically, the optimized emission magnitudes fall within the uncertainty range defined by the multi-inversion ensemble. The derived spatial distribution is also broadly similar to that obtained for the bae-case adjoint inversion (Fig. S12 vs. Fig. 7).

Finally, given findings pointing to inventory underestimates of US oil and gas emissions (Alvarez et al., 2018; Barkley et al., 2019; Gvakharia et al., 2017; Peischl et al., 2016), we tested whether a prior bias for this source could be aliasing our emission estimates. Specifically, we performed sensitivity inversions (sector-based and GMM) with the prior fossil fuel emissions doubled. Two main results emerge. First, the derived livestock emission SFs change by <12% from the base-case, while those for wetlands (excluding winter) change by <4%. Second, the "other" source category (encompassing fossil fuels) remains close to the prior under both base-case and doubled fossil fuel scenarios. We conclude that i) our wetland and livestock estimates are not strongly sensitive to fossil fuel-related emission errors, and ii) the derived oil and gas fluxes are prior-dependent and only weakly constrained by the GEM observing system.

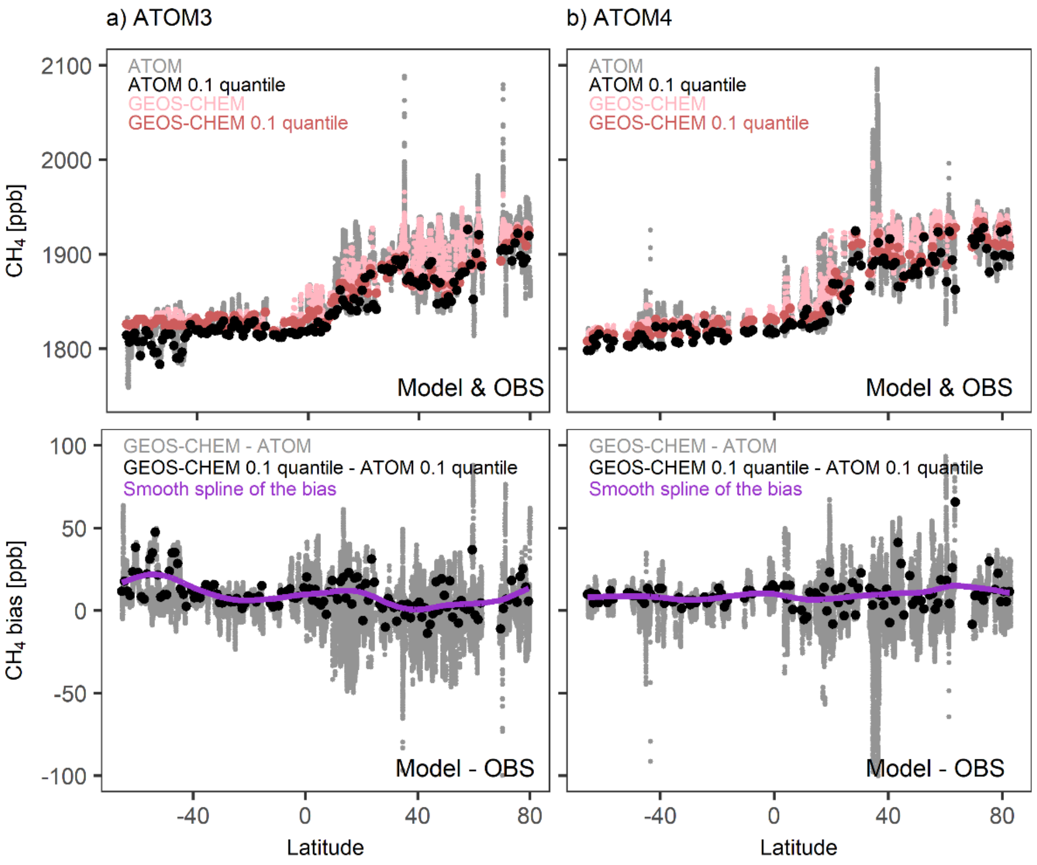

Figure S1. Correction of the model methane background based on aircraft measurements from ATom3 (left column) and ATom4 (right column). Plotted in the top panels are the mean (pink) and 0.1 quantile (red) observed tropospheric methane mixing ratios by one-degree latitude bins, along with the corresponding model values (grey and black). Bottom panels show the resulting model-measurement mismatches with a smooth spline fit to the 0.1 quantile difference.

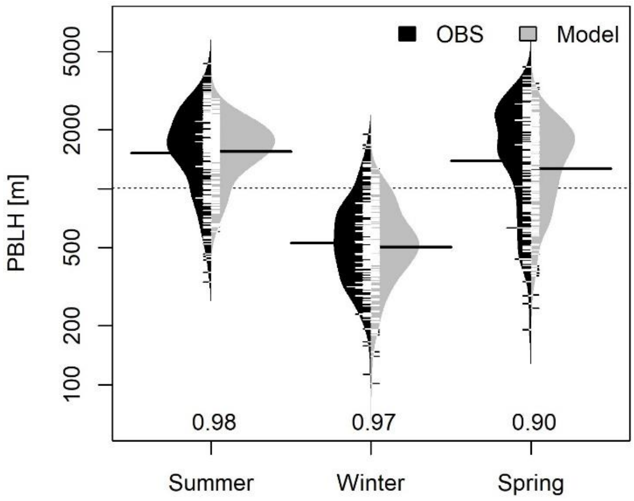

Figure S2. Model:measurement planetary boundary layer height (PBLH) comparison. The bean-plots compare radiosonde-based (00:00 UTC, 18:00 or 19:00 local time) and GEOS-FP average midday (12:00-16:00 local time) PBLH values for summer (Aug. 2017, GEM1), winter (Jan. 2018, GEM2) and spring (May 2018, GEM3). Each bean displays density distributions for the radiosonde (black) and GEOS-FP (grey) data, along with the individual datapoints (short lines) and their mean values (black lines). The mean model:measurement ratios for each season are indicated above the x-axis. Comparisons include 168, 176, and 170 total data points in summer, winter, and spring respectively.

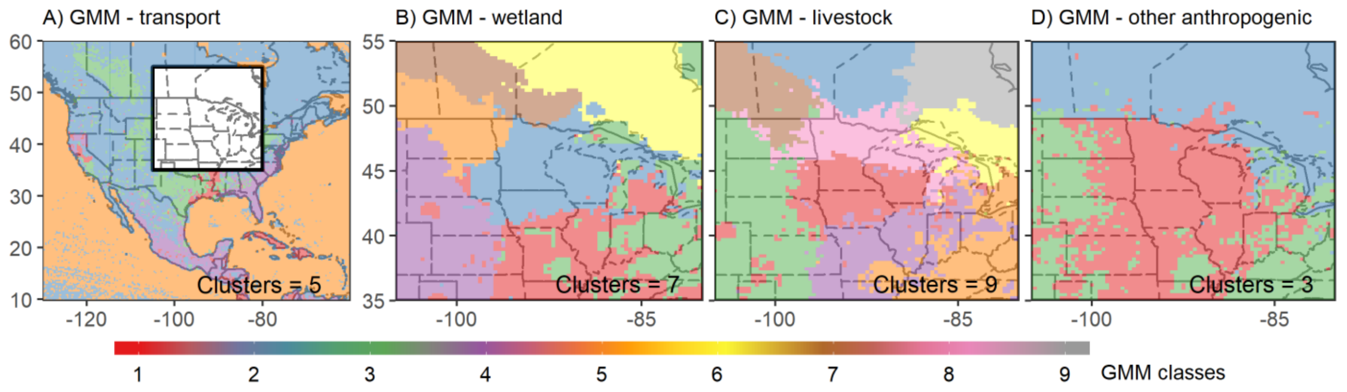

Figure S3. GMM clusters used for methane source optimization. As detailed in-text these include clusters representing transported emissions from outside the Upper Midwest domain (panel A), as well as Upper Midwest wetland (panel B), livestock (panel C), and other anthropogenic (panel D) emissions. Not plotted are clusters (1 each) for Upper Midwest oil + gas + coal, rice, biomass burning, and other natural emissions.

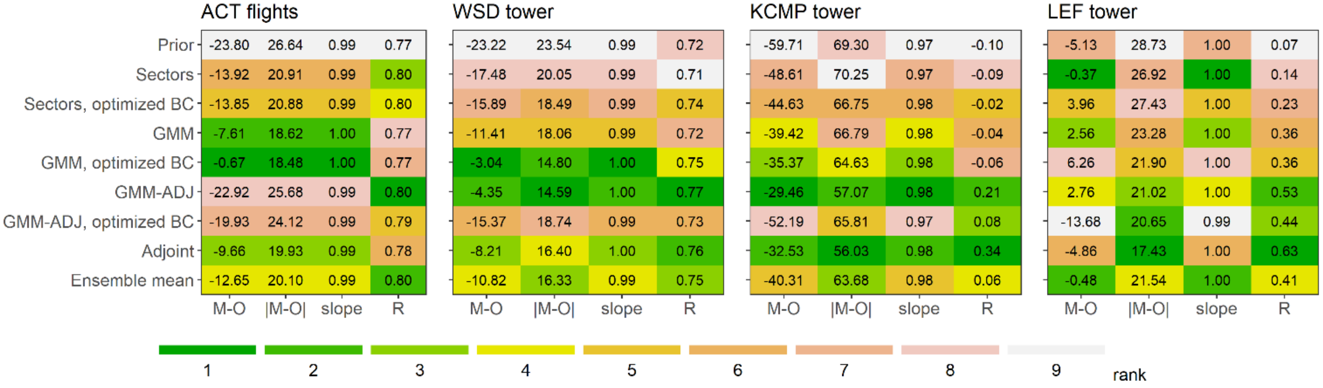

Figure S4. Same as Fig. 4, but using independent measurements from the same timeframe as the GEM flights. See Sect. 2.4 for details.

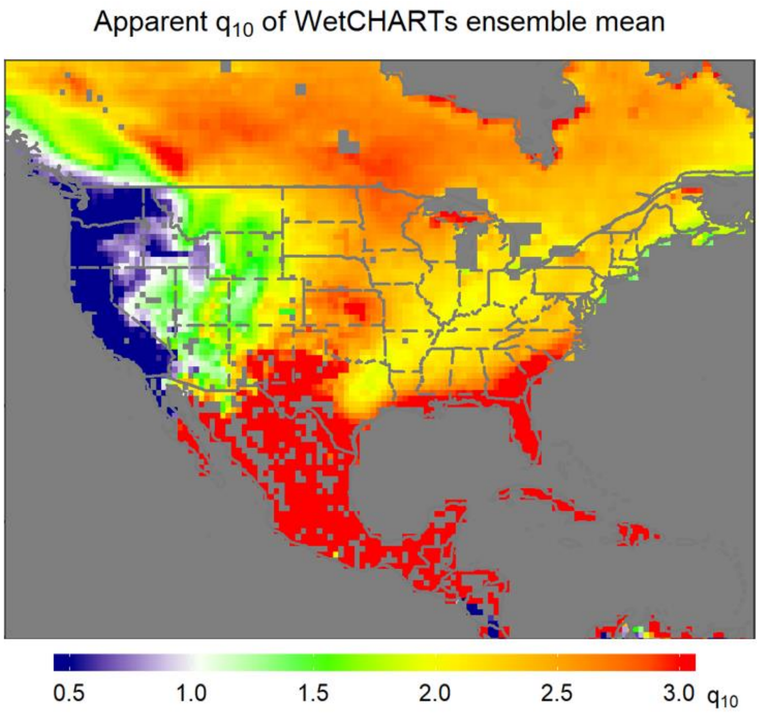

Figure S5. Net temperature dependence of methane emissions (CH_4:T q_{10}) for the WetCHARTs ensemble mean. Results are computed from the WetCHARTs methane emissions and surface skin temperatures from ERA-Interim (ECMWF, 2019).

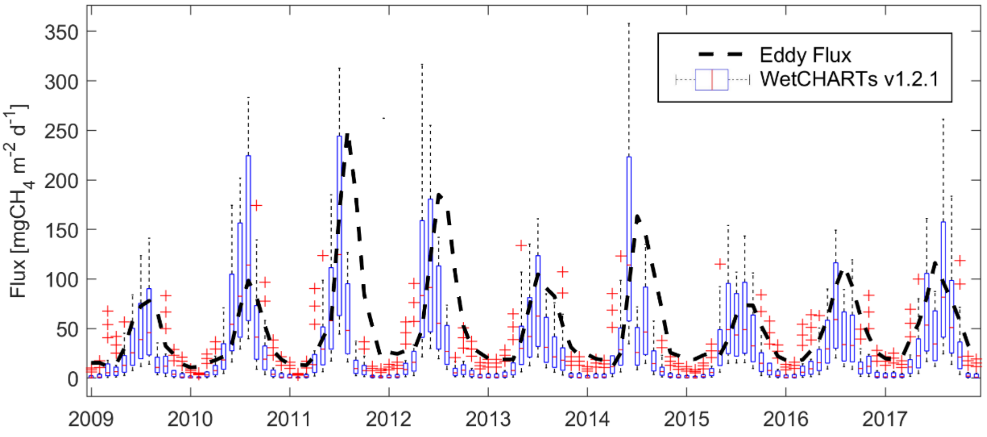

Figure S6. Monthly mean wetland fluxes at the Minnesota Bog Lake peatland during 2009-2017. Eddy covariance flux measurements (black; (Deventer et al., 2019)) are compared to WetCHARTs predictions (blue). Boxes and whiskers encompass the interquartile range (IQR) and full range, respectively, across the WetCHARTs extended ensemble (excluding outliers). Outliers (defined as points exceeding the 0.25 or 0.75 quantile by >1.5× the IQR) are plotted separately in red.

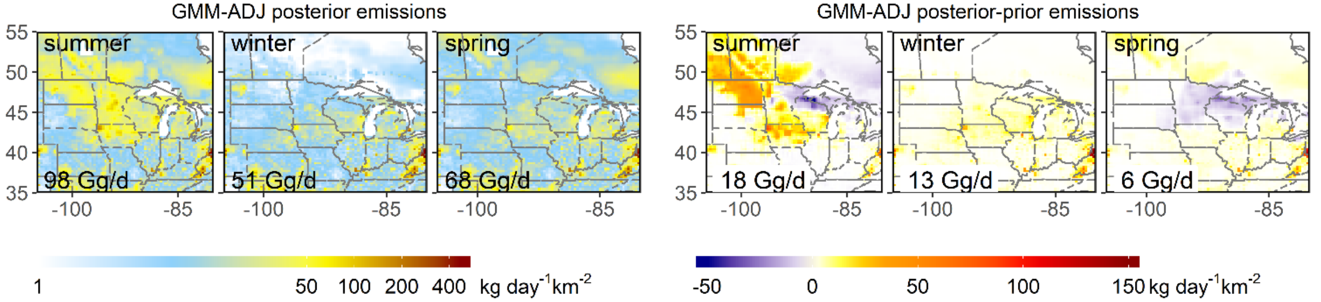

Figure S7. Same as Fig. 7, but showing results for the GMM-ADJ inversion.

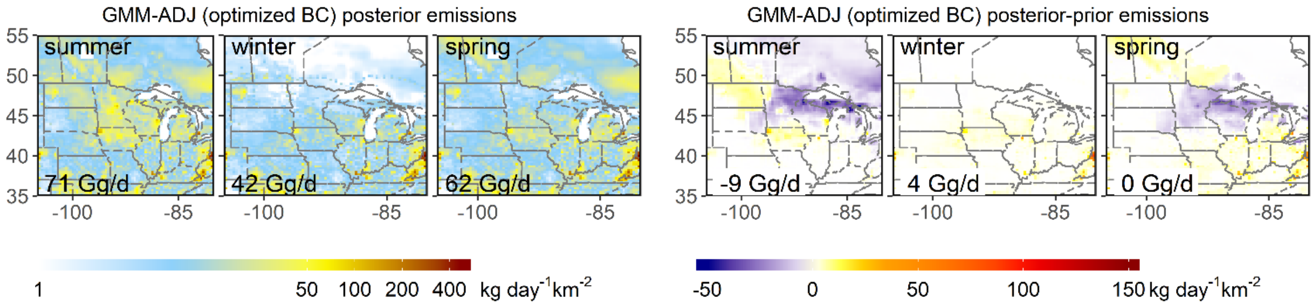

Figure S8. Same as Fig. 7, but showing results for the GMM-ADJ inversions with boundary condition optimization.

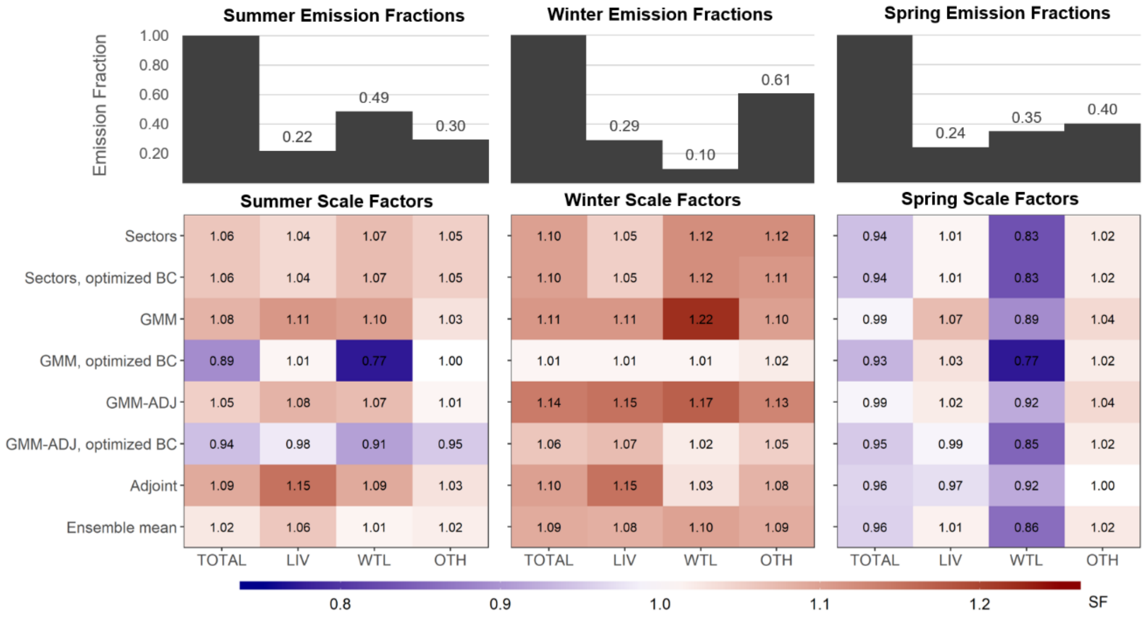

Figure S9. Same as Fig. 3 but using regulation parameter $\gamma = 1$.

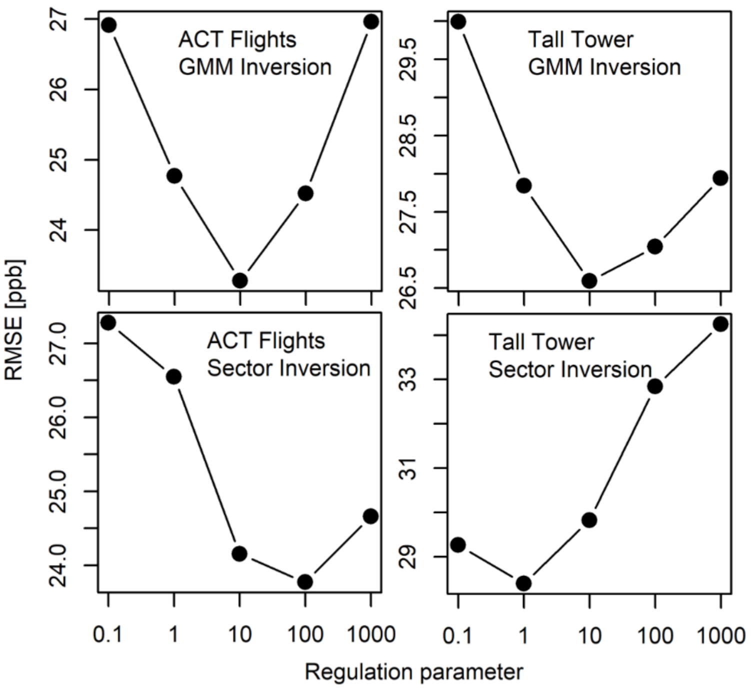

100 **Figure S10.** Aggregated level of agreement between optimized model results and independent measurements as a function of regularization parameter ($\gamma \in [0.1, 1000]$). Plots show the root mean square error (RMSE) for the analytical (i.e., sector-based and GMM) inversion results with respect to observations from the ACT-America campaign and the KCMP, LEF, and WSD tall towers. $\gamma = 10$ is selected as the best case.

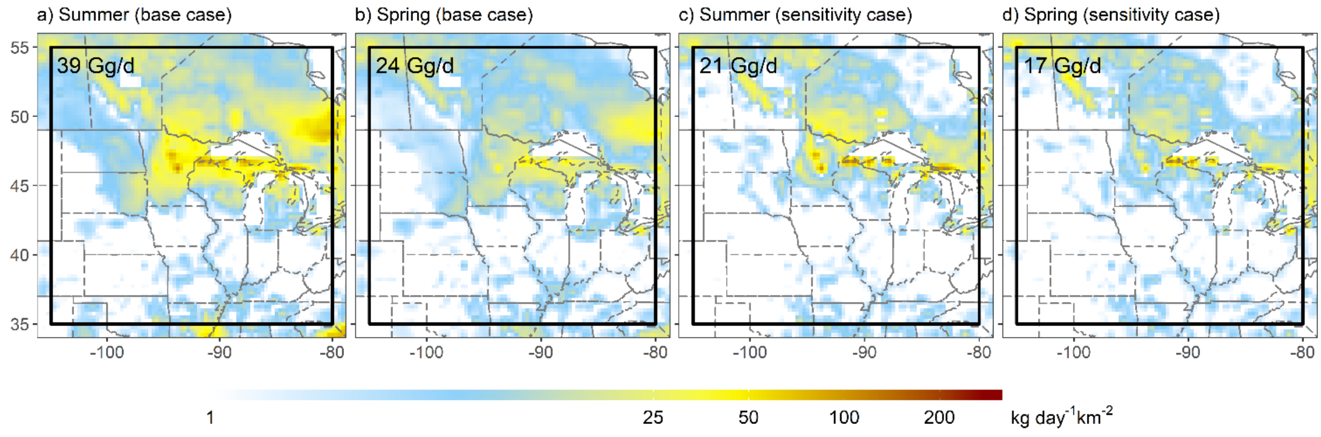

Figure S11. Prior wetland emissions of a-b) base cases (WetCHARTs ensemble mean) and c-d) sensitivity cases (an individual WetCHARTs member) for summer and spring.

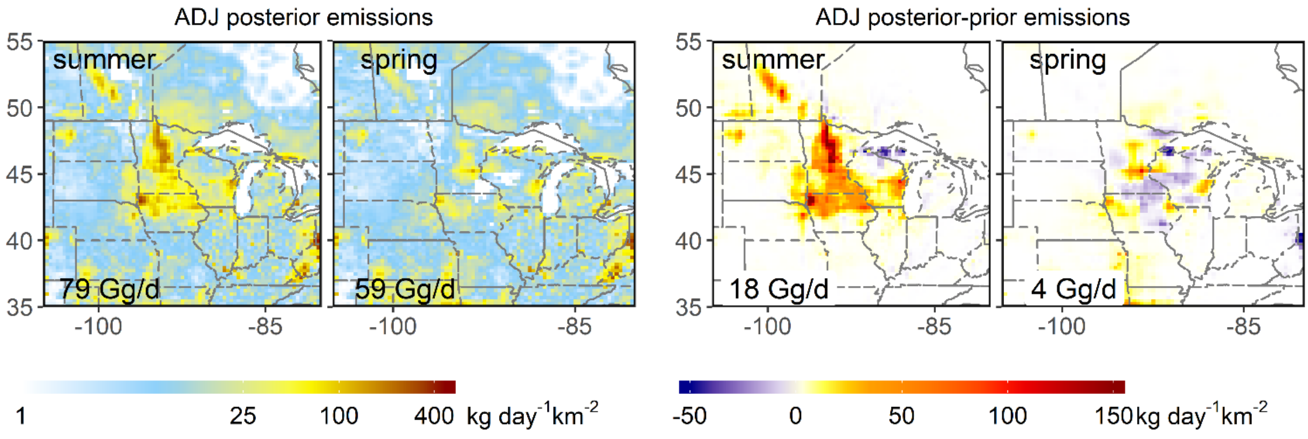

110 **Figure S12.** Same as Fig. 7, but showing results using an alternate wetland emission estimate as prior (see Sect. S1) Panels on the right reflect the difference between the optimized emissions and the alternate prior emissions.

SI References

Alvarez, R. A., Zavala-Araiza, D., Lyon, D. R., Allen, D. T., Barkley, Z. R., Brandt, A. R., Davis, K. J., Herndon, S. C., Jacob, D. J., Karion, A., Kort, E. A., Lamb, B. K., Lauvaux, T., Maasakkers, J. D., Marchese, A. J., Omara, M., Pacala, S. W., Peischl, J., Robinson, A. L., Shepson, P. B., Sweeney, C., Townsend-Small, A., Wofsy, S. C., and Hamburg, S. P.: Assessment of methane emissions from the U.S. oil and gas supply chain, Science, 361, 186-188, 10.1126/science.aar7204, 2018.

Barkley, Z. R., Davis, K. J., Feng, S., Balashov, N., Fried, A., DiGangi, J., Choi, Y., and Halliday, H. S.: Forward modeling and optimization of methane emissions in the South Central United States using aircraft transects across frontal boundaries, Geophysical Research Letters, 46, 13564-13573, 10.1029/2019gl084495, 2019.

Deventer, M. J., Griffis, T. J., Roman, D. T., Kolka, R. K., Wood, J. D., Erickson, M., Baker, J. M., and Millet, D. B.: Error characterization of methane fluxes and budgets derived from a long-term comparison of open- and closed-path eddy covariance systems, Agricultural and Forest Meteorology, 278, 107638, 10.1016/j.agrformet.2019.107638, 2019.

ECMWF: ERA-Interim, 2019. https://www.ecmwf.int. (access in June 2020).

Gvakharia, A., Kort, E. A., Brandt, A., Peischl, J., Ryerson, T. B., Schwarz, J. P., Smith, M. L., and Sweeney, C.: Methane, black carbon, and ethane emissions from natural gas flares in the Bakken Shale, North Dakota, Environmental Science & Technology, 51, 5317-5325, 2017.

Peischl, J., Karion, A., Sweeney, C., Kort, E., Smith, M., Brandt, A., Yeskoo, T., Aikin, K., Conley, S., and Gvakharia, A.: Quantifying atmospheric methane emissions from oil and natural gas production in the Bakken shale region of North Dakota, Journal of Geophysical Research: Atmospheres, 121, 6101-6111, 2016.
